# Quantitative Proteomic Analysis of Germination of *Nosema bombycis* Spores under Extremely Alkaline Conditions

**DOI:** 10.3389/fmicb.2016.01459

**Published:** 2016-09-21

**Authors:** Han Liu, Bosheng Chen, Sirui Hu, Xili Liang, Xingmeng Lu, Yongqi Shao

**Affiliations:** Laboratory of Invertebrate Pathology, College of Animal Sciences, Zhejiang UniversityHangzhou, China

**Keywords:** proteome, germination, *Nosema bombycis*, microsporidia, metabolic pathway

## Abstract

The microsporidian *Nosema bombycis* is an obligate intracellular pathogen of the silkworm *Bombyx mori*, causing the epidemic disease Pebrine and extensive economic losses in sericulture. Although *N. bombycis* forms spores with rigid spore walls that protect against various environmental pressures, ingested spores germinate immediately under the extremely alkaline host gut condition (Lepidoptera gut pH > 10.5), which is a key developmental turning point from dormant state to infected state. However, to date this process remains poorly understood due to the complexity of the animal digestive tract and the lack of genetic tools for microsporidia. Here we show, using an *in vitro* spore germination model, how the proteome of *N. bombycis* changes during germination, analyse specific metabolic pathways employed in detail, and validate key functional proteins *in vivo* in silkworms. By a label-free quantitative proteomics approach that is directly based on high-resolution mass spectrometry (MS) data, a total of 1136 proteins were identified with high confidence, with 127 proteins being significantly changed in comparison to non-germinated spores. Among them, structural proteins including polar tube protein 1 and 3 and spore wall protein (SWP) 4 and 30 were found to be significantly down-regulated, but SWP9 significantly up-regulated. Some nucleases like polynucleotide kinase/phosphatase and flap endonucleases 1, together with a panel of hydrolases involved in protein degradation and RNA cleavage were overrepresented too upon germination, which implied that they might play important roles during spore germination. The differentially regulated trends of these genes were validated, respectively, by quantitative RT-PCR and 3 proteins of interest were confirmed by Western blotting analyses *in vitro* and *in vivo*. Furthermore, the pathway analysis showed that abundant up- and down-regulations appear involved in the glycolysis, pentose phosphate pathway, purine, and pyrimidine metabolism, suggesting preparations of energy generation and substance synthesis for the following invasion and proliferation inside the host. This report, to our knowledge, provides the first proteomic landscape of *N. bombycis* spores, and also a stepping stone on the way to further study of the unique infection mode of this economically important pathogen and other microsporidia in general.

## Introduction

Microsporidia are a type of obligate intracellular parasite related to fungi that include over 1500 species in ~187 genera (Vavra and Lukes, 2013). Microsporidia are important pathogens that infect a wide variety of animal groups, including vertebrates and invertebrates (Bhat et al., 2009). For instance, *Nosema bombycis*, the first reported microsporidian species, can cause a highly mortal disease referred to as pebrine in the silkworm *Bombyx mori*. Infecting through horizontal and vertical transmissions, *N. bombycis* proliferates continuously inside the host, which leads to chronic damage to all of larval tissues and organs including the intestines, silk glands, muscles, and malpighian tubules, and ultimately death. Thereby, *N. bombycis* represents a significant threat to sericulture worldwide (Becnel and Andreadis, 1999).

When surviving outside of the host, microsporidia form environmentally resistant spores that are protected by a thick two-layered wall, and are able to maintain metabolic activity (Vavra and Larsson, 1999). These mature spores, which are not germinated, are considered dormant spores. Upon coming into contact with host cells or being triggered by appropriate stimuli, dormant spores rapidly start the germination process. They fire a special invasion organelle, known as the polar tube, to penetrate the host cell membrane, and transfer the infective sporoplasm into the host cytoplasm, where proliferation and the next round of spore production occurs (Schottelius et al., 2000). As the first step in the microsporidia invasion, biological changes during the spore germination process have been suggested to play important roles (Troemel and Becnel, 2015). However, to date this process still remains poorly understood due to complex host-pathogen interactions and the lack of genetic tools for microsporidia.

By establishing a method for the *in vitro* spore germination, the polar tube firing process and invasion of the host cell have been observed in several microsporidian species (Troemel and Becnel, 2015). It has been broadly accepted that an appropriate stimulus such as pH, ionic species, osmotic pressure, or radiation can make microsporidian spores germinate, though the exact mechanism has not been fully elucidated.

A number of spore wall proteins (SWPs) have been identified and confirmed to be related to germination and invasion (Zhao W. et al., 2015). SWP5 has been confirmed to have the ability to protect spores from phagocytic uptake by cultured insect cells to modulate host cell invasion and interact with the polar tube (Cai et al., 2011; Li et al., 2012). An IG-like protein denoted BmTLP from *B. mori* has been proven to interact with SWP26, which is involved in endospore formation, host cell adherence, and infection *in vitro* (Li et al., 2009; Zhu et al., 2013). SWP16 was identified as a new exospore protein and is probably involved in *N. bombycis* spore adherence (Wang et al., 2015). However, the protein regulation mechanism of spore germination process is still largely unknown.

With the rapid development of proteomics technologies in combination with increased genome sequence information, proteomic analysis has become one of the most important strategies for characterizing expression profiles and pathway changes in diverse biological systems. Using traditional two-dimensional electrophoresis combined with mass spectrometry (MS), 80 proteins were found to be abundantly changed upon horsetail (*Equisetum arvense* L.) spore germination (Zhao Q. et al., 2015). Similarly, enzymes that are crucial to stress response were identified by this approach in thermophilic Archaea *Thermococcus kodakarensis* KOD1 (Jia et al., 2015). Recently, a promising label-free quantitative (LFQ) proteomic approach has been developed, in which peptide intensities measured during individual liquid chromatography (LC) runs are compared across runs (Tao et al., 2013). The advantage of this method is that it can be applied to any proteomic sample directly.

In the current study, we provides the first example of using label-free quantitation to investigate and compare the global proteomic changes during *N. bombycis* spore germination, which is regarded as the first step in host invasion. A total of 1136 proteins were identified with high confidence, with 127 proteins being significantly changed. Based on these data, proteins involved in germination and invasion were identified, as well as a number of important changes in metabolic pathways during the germination process. An in-depth understanding of spore germination could reveal useful insights into the unique mode of infection of this economically important pathogen and other microsporidia in general.

## Materials and methods

### Insect sample collection and PH measurements

The silkworms were provided by the Silkworm Germplasm Bank at the College of Animal Sciences, Zhejiang University, and reared under standard conditions (25° ± 1°C, 70 ± 5% humidity with a photoperiod of 14 h of light and 10 h of dark) using fresh mulberry leaves. The 5th instar larvae were anesthetized on ice for 30 min and dissected carefully. A pH microelectrode (Unisense, Denmark) with a tip diameter of 20–30 μm was calibrated with standard buffers at pH 4.0, 7.0, and 10.0 and directly applied to the freshly extracted guts as previously described (Sudakaran et al., 2012). The pH was recorded in three replicates in different gut regions.

### Spore purification and *in vitro* germination model

*N. bombycis* spores were propagated and purified from infected silkworms as previously described (Mei and Jin, 1989; Zhang et al., 2007). Firstly, 3rd instar molted larvae were fed mulberry leaves that were artificially contaminated by *N. bombycis* spores (~3 × 10^4^ spores per larva). Heavily infected silk glands and midguts were collected from 5th instar larvae at the fifth day and gently homogenized in physiological saline with a glass homogenizer. Large silkworm debris was removed by filtrating twice through four layers of cheesecloth. After centrifugation (3000 g, 5 min), the sediment was resuspended in 20 mL of a solution containing 0.05% saponin and 0.05% (v/v) Triton X-100 in phosphate buffered saline (PBS). The cell suspension was passed through a plastic syringe with an inserted glass wool wad. A crude spore suspension was obtained by differential centrifugation after most of the contaminant was removed. The spores were further purified through continuous Percoll (pH = 7.2, GE Healthcare, Chicago, UK) gradient centrifugation (46,000 g, 60 min). After washes with distilled water, the spore purity was examined by phase-contrast microscopy. A total of 1 × 10^9^ purified spores were divided into two parts on average and washed three times with PBS. A non-germinated spore (NGS) control sample was then treated with 200 μL of physiological saline. The germinated spores (GS) sample was treated with 200 μL of GKK germination buffer (0.05 M Glycine, 0.05 M KOH, and 0.375 M KCl, pH = 10.5), and both the NGS and GS samples were incubated at 30°C for 50 min. The experiment was performed for five independent biological replicates with the control and treatment groups.

### Protein isolation and trypsin digestion via the FASP method

After germination, all 10 samples were then separately lysed with 1 mL of SDT-lysis buffer (4% SDS, 0.1 mol/L DTT, and 0.1 mol/L Tris-HCl, pH = 7.6) and 10 μL Protease Inhibitor Cocktail (Sangon, Shanghai, China) using acid-washed glass beads (diameter: 425–600 μm; Sigma, St Louis, USA) in a Precellys-24 (Bertin Technologies, Aix en Provence, France). The mixture was broken for 20 s on ice with 30 s intervals for 5 times and centrifuged at 14,000 g at 4°C. The protein concentration was detected by a Bicinchoninic Acid (BCA) Protein Assay Kit (Beyotime, Haimen, China). Fifty micrograms of each sample was digested by the FASP procedure (Wisniewski et al., 2010). DTT was added to the sample to a final concentration of 0.1 mol/L, and the mixture was incubated at 95°C for 5 min. Then, after adding 200 μL of UA buffer (8 M urea and 0.1 M Tris-HCl, pH = 8.5), each sample was concentrated in 30k Microcon filtration devices (Millipore, Boston, USA) and centrifuged at 14,000 g for 15 min at 20°C. Then, another 200 μL of UA buffer was added and the samples were centrifuged again at 14,000 g for 15 min. This step was repeated once. Next, the concentrate was mixed with 100 μL of 50 mM/L iodoacetamide (IAA) in UA buffer and incubated for an additional 20 min at room temperature in the dark. After that, the IAA was removed by centrifugation at 14,000 g for 15 min. One-hundred microliters of 50 mM/L NH_4_HCO_3_ was added and the samples were centrifuged at 14,000 g for 15 min; this step was repeated twice. Finally, 50 μL of 50 mM/L NH_4_HCO_3_, and trypsin (1:50, enzyme to protein) was added and incubated with the mixture at 37°C for 16 h. The tryptic peptide mixtures were collected for further analysis.

### LC-MS/MS

Reverse phase-high performance liquid chromatography (RP-HPLC) separation was achieved on an Easy-nLC nano-LC system (Thermo Fisher Scientific, Bremen, Germany) equipped with a self-packed column (75 μm × 150 mm; 3 μm ReproSil-Pur C18 beads, 120 Å, Dr. Maisch GmbH, Ammerbuch, Germany) at a flow rate of 250 nL/min. The RP-HPLC mobile phase A was 0.1% formic acid in water and mobile phase B was 0.1% formic acid in acetonitrile. The peptides were eluted using a gradient (2–80% mobile phase B) over a 3 h period. A nano-ESI Q-Exactive mass spectrometer (Thermo Fisher Scientific, Bremen, Germany) operated in data-dependent mode. The mass spectrometer was set such that each full MS scan followed by MS/MS for the 10 highest-intensity ions with the following parameters: precursor ion charges ≥ +2, a precursor ion isolation window of 2 Da, and a normalized collision energy of 30 for HCD. Dynamic Exclusion™ was set at 25 s. The full mass and subsequent MS/MS analyses were performed in an Orbitrap analyzer with *R* = 70,000 and *R* = 17,500 (at m/z 200), respectively. Five biological replicates were performed with NGS and GS.

### Data analysis

The MS data were analyzed using the MaxQuant software (http://maxquant.org/, version 1.3.0.5). Carbamidomethyl (C) was set as a fixed modification, while oxidation (M, +15.99492 Da) was set as a variable modification. Proteins were identified by searching the MS and MS/MS data for peptides against the *N. bombycis* protein database from NCBI (http://www.ncbi.nlm.nih.gov/). Trypsin/P was selected as the digestive enzyme with two potential missed cleavages. Label-free quantification of NGS and GS was performed in MaxQuant as previously described (Cox et al., 2009). Protein abundance was calculated on the basis of the normalized spectral protein intensity (label-free quantitation intensity, LFQ intensity). The *p*-value of Log2LFQ intensity of each protein between the NGS and GS by two-tailed Student's *t*-test using the Perseus program, and *p* < 0.05 was set as a cutoff criterion for a significant change. The functional annotations of the identified proteins were initially assigned using the Blast2Go program (Conesa et al., 2005). BLASTp searches were first performed against the NCBI protein database and further analyses included Gene Ontology (GO) and Enzyme Code (EC) annotations. To investigate the significantly changing protein patterns in specific pathways, the Blast2Go program was used to locate the proteins in the Kyoto Encyclopaedia of Genes and Genomes (KEGG) pathway database (http://www.genome.jp/kegg; Conesa et al., 2005; Gotz et al., 2008). Briefly, all the amino acid sequences of 1136 identified proteins and 127 significantly changed proteins were firstly input into the Blast2Go program, respectively, then the mapping from KEGG database (online) was performed and the metabolic pathways of identified proteins were obtained.

### RNA extraction and validation by quantitative RT-PCR analysis

After *in vitro* germination, the NGS and GS were separately lysed with 1 ml of TRIzol reagent (Invitrogen, Carlsbad, USA) using acid-washed glass beads in a Precellys-24. The total RNA from the broken spores was extracted according to the TRIzol Reagent manufacturer's protocol. cDNA synthesis for NGS and GS was performed using the PrimeScript™ RT Reagent Kit with gDNA Eraser (TaKaRa, Dalian, China). To validate the results of the label-free quantitation, quantitative real-time PCR (qRT-PCR) was performed for 16 randomly selected genes with significant changes in protein levels. Sixteen primers were designed using the Primer5 software (Premier Biosoft International) and all of the primer sequences were listed in Table S1. The internal control is β-tubulin. Quantitative real-time PCR (qRT-PCR) experiments were performed using the Roche LightCyler 480 system (Roche, Basel, Switzerland). Each reaction was conducted with a volume of 20 μL using LightCycler 480 SYBR Green 1 Master (Roche, Basel, Switzerland). All of the assays for a particular gene were performed with triplicate biological replicates and technical replicates. The expression level of each target gene was normalized to the level of β-tubulin. The fold change of each gene was calculated by the 2^−ΔΔCT^ method (Schmittgen and Livak, 2008) and determined by three independent quantitative PCR amplifications of RNA that had been independently extracted.

### Western blot analysis and enzyme activity assay

Mulberry leaves (2.5 × 2.5 cm) were firstly disinfected by bleaching powder and artificially contaminated with *N. bombycis* spores. 8 × 10^7^ spores were fed to each 5th instar larva (similar body weight). The larvae of “0 h” treatment group were collected when the larvae ingested mulberry leave completely, the larvae of “1” and “2 h” treatment group were collected at 1 and 2 h post-mulberry leaves were ingested, respectively. Then the spore protein of each treatment group was prepared. Briefly, the midgut was pulled out from the larvae to the ice cold PBS. The midgut and spores inside were lysed with 1 mL of SDT-lysis buffer and 10 μL Protease Inhibitor Cocktail (Sangon, Shanghai, China) using acid-washed glass beads in a Precellys-24 tissue homogenizer. The mixture was broken for 25 s on ice with 30 s intervals for 9 times and centrifuged at 14,000 *g* at 4°C. The total protein of “0,” “1,” and “2 h” were collected and protein concentration was determined using the BCA method.

Total Proteins of NGS, GS, “0,”, “1,” and “2 h” were resolved in 12% SDS-PAGE and transferred onto nitrocellulose membrane, blocked with 5% non-fat milk (in PBS), and incubated with the respective primary antibodies and beta-Actin (as a control for equal protein loading, HuaAn Biotechnology Company, Hangzhou, China) overnight at 4°C. The rabbit anti-SWP4 polyclonal antibody (diluted in 1:2000), rabbit anti-Flap endonucleases 1 polyclonal antibody (diluted in 1:2000) and rabbit anti-Pnkp polyclonal antibody (diluted in 1:2000) were produced by Genscript Biotechnology Company and the antigen sequence for respective antibody preparation were “CYVMKKKLKKSARKK,” “KENSKKGIVERPLSC,” and “CDKIPFMFDDSNFN,” respectively. The immunoblot detection was carried out using an ECL Western blotting analysis system using peroxidase conjugated secondary antibodies (Sigma, St Louis, USA). Every gel was loaded with molecular weight markers including proteins with molecular weight from 10 to 170 kDa (Bio-Rad, Hercules, USA).

Protease activity was measured using a commercial protease assay kit (Zhang et al., 2015). For NGS, purified spores (1 × 10^10^) were treated with 250 μL of physiological saline, while GKK germination buffer was used for GS. Both NGS and GS were incubated at 30°C for 30 min. Then, the supernatants were collected and the protein concentration was determined using the BCA method. Protease activities in two groups were measured by a Protease Assay™ Kit (G-Biosciences, St. Louis, USA), which uses a dye-labeled protein substrate. Briefly, 20 μL sample supernatant, 2.5 μL protease substrate solution, and 27.5 μL incubation buffer were mixed in a micro tube and incubated at 37°C for 24 h. After incubation, 50 μL precipitation agent was added in the tube and incubated at 37°C for 10 min. Then the samples were centrifuged at 12,000 *g* for 5 min and 80 μL supernatant was transferred to clean tubes with 120 μL assay buffer. The reaction was recorded at 570 nm against the blank. Chemically stabilized trypsin (MSG-Trypsin™) was supplied in the kit for preparing a general protease activity standard curve. The protease activity is proportional to the intensity of the reaction color, which can be quantified using a standard calibration curve.

## Results

### The extremely alkaline gut environment of the silkworm stimulates the germination of *N. bombycis* spores

**Figure 1:**
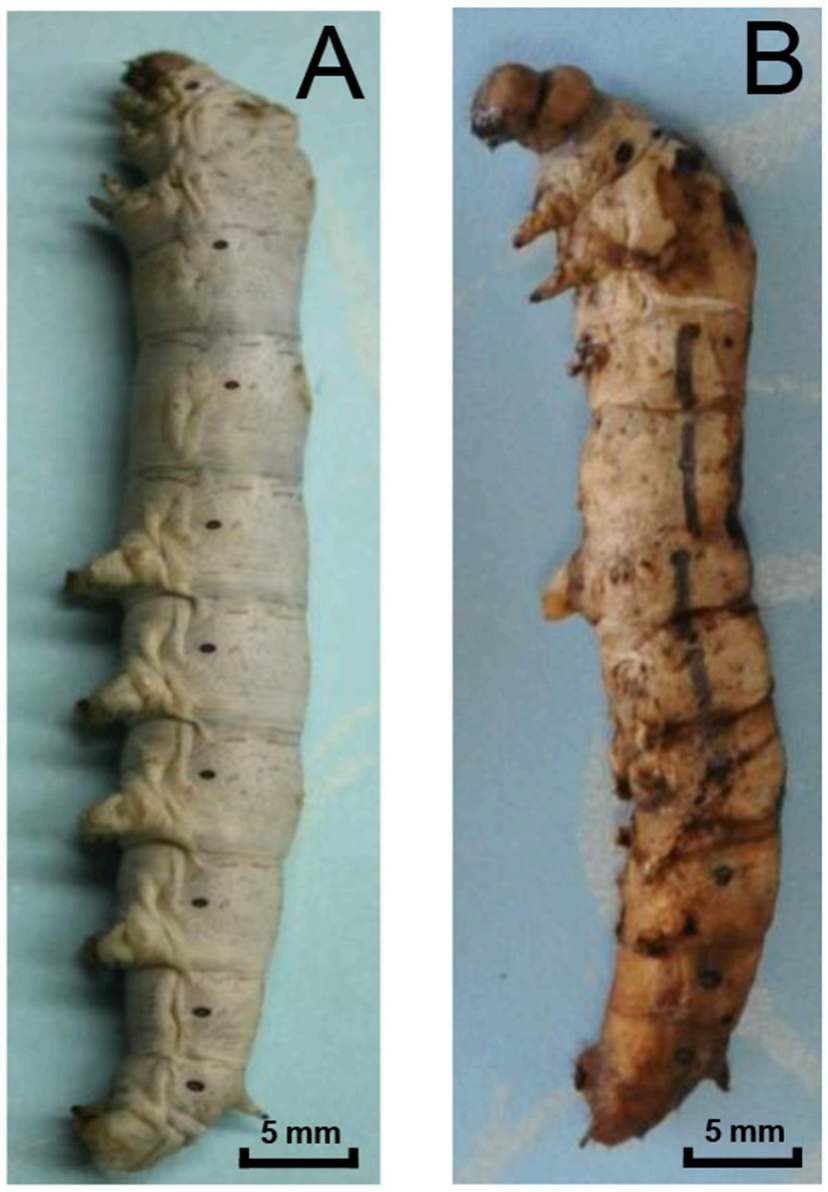
**Pebrine disease of silkworms caused by *N. bombycis*. (A)** A normal 5th instar silkworm. **(B)** Infected larvae show typical symptoms of disease: they are usually covered in brown or black dots and are unable to spin silkworm thread.

*N. bombycis* spores generally enter the silkworm host through the esophagus and are carried to the midgut where they germinate rapidly to deliver the germ cell (sporoplasm) into the host cell to initiate intracellular infection, which cause the pebrine disease in silkworm. After infections, larvae are inactive and slow in development. Later brown or black spots, a characteristic disease symptom, appear throughout their bodies and lead to death (Figure 1).

**Figure 2:**
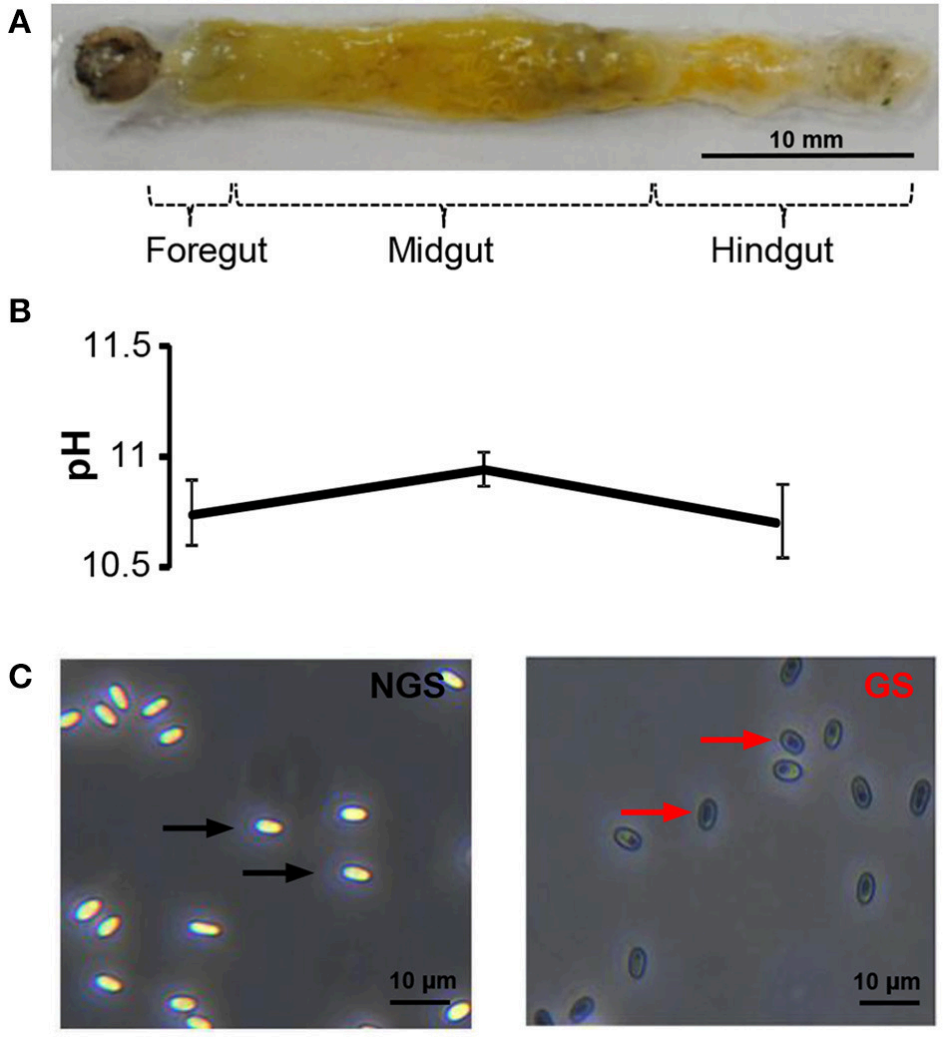
**The spore germination model of *N. bombycis*. (A)** Dissected alimentary canal from a normal 5th instar larva of *B. mori* indicating the gut segmentation. **(B)** Extremely high pH in the gut lumen. The pH was measured in each gut region and data are shown as mean ± S.E.M. of at least three biological replicates. **(C)** Comparison between non-germinated (left) and germinated spores (right) in the *N. bombycis* spore germination model. *N. bombycis* spores were treated with GKK buffer (pH = 10.5) to initiate germination (polar tube firing) and their morphology were analyzed using phase-contrast microscopy. Black arrows indicate non-germinated spores (NGS); red arrows indicate germinated spores (GS). The scale bar represents 10 μM.

The midgut of silkworm, just like that of other herbivorous lepidopteran larvae, is a simple tube comprising the largest part of the whole body (Figure 2A). And midgut conditions are highly complex with various digestive enzymes, ion, and pH shifts, which might impact the *N. bombycis* spore germination. Among those factors, the extremely high pH is particularly noticeable. The silkworm midgut lumen provides a maximum pH of 11 *in situ* (Figure 2B), among the highest pH-values known to be generated by any biological system (Dow, 1992). The longitudinal pH profile observed in the gut region from anterior to posterior revealed that the pH rises to a peak in the middle region and declines again in the posterior region (Figure 2B).

To test the contribution of pH on spore germination, *N. bombycis* spores were treated with a GKK germination buffer with a pH of 10.5 to induce germination (polar tube firing). Under phase-contrast microscopy, the non-germinated spores (NGS) and germinated spores (GS) were observed according to their unique developmental morphology. The bright and smooth spores (Figure 2C, black arrow) were non-germinated, whereas the dark spores (Figure 2C, red arrow) were germinated. Therefore, this simplified *in vitro* spore germination model successfully initiates the spore germination process and was employed to obtain the total protein from NGS and GS of *N. bombycis* for further proteomic investigation.

### Label-free quantitative (LFQ)proteomic analysis of NGS and GS of *N. bombycis*

**Figure 3:**
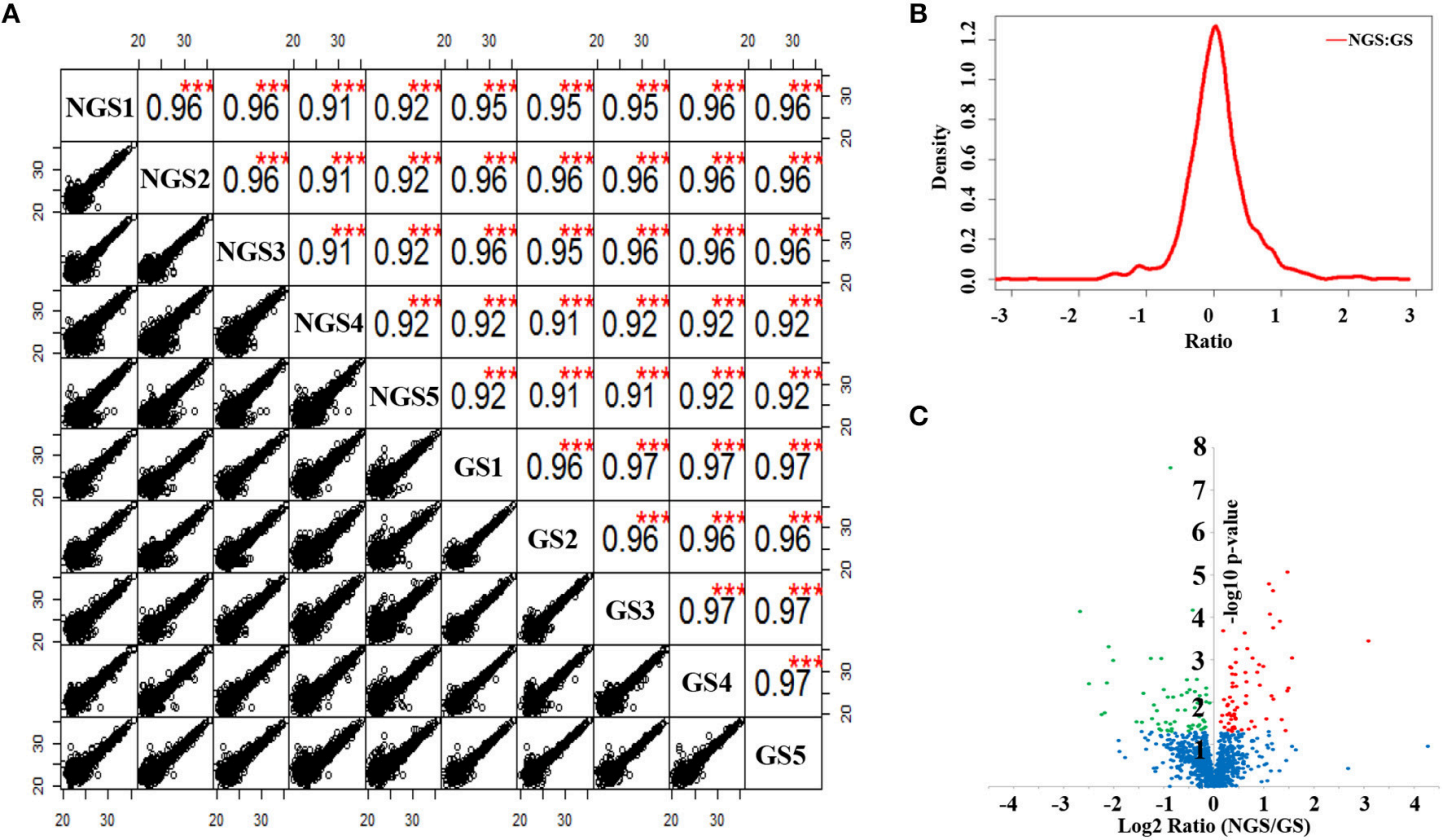
**Label-free quantitative proteomic analysis of *N. bombycis* NGS and GS. (A)** Scatter plots and correlations between samples. **(B)** Density plot of the ratio between NGS and GS. **(C)** Volcano plot of the 1136 identified proteins between NGS and GS. A total of 127 proteins were identified as significantly changed proteins induced by germination, all of which exhibited a *p* < 0.05. Red represents the significantly up-regulated proteins, green the significantly down-regulated proteins, and blue the proteins that did not change significantly during spore germination. ^***^p < 0.01.

The total protein extracted from the NGS group and GS group of *N. bombycis* were analyzed in parallel using a LFQ proteomic approach. The relative LFQ intensities of the proteins across each individual samples were calculated using MaxQuant algorithms. The mass tolerance for peptide precursor ions and product ions was set at 6 and 20 ppm, respectively, and the false discovery rates at both peptide and protein levels were controlled to be <1%. These stringent filtration criteria provided highly confident protein identification. The *p*-value of Log2LFQ intensity of each protein between the NGS and GS groups were calculated using Student's *t*-test. As shown in the Figure 3A, the LFQ was highly reproducible between the five biological replicates in each treatment group and between the LC-MS/MS runs from the different treatment groups. The correlation between the normalized LFQ intensities was higher than 0.9 for each comparison of samples. The density plot of the log_2_ ratio between NGS group and GS group are similar to normal distribution (Figure 3B), which indicated that the experimental procedure was performed without systematic bias toward different samples. It thus allowed an accurate quantitative comparison of the protein expression in *N. bombycis* before and after the spore germination. After searching a non-redundant predicted protein dataset of *N. bombycis*, 1136 proteins were identified from analysis of the entire 10 LC-MS/MS raw data files of two treatment groups. The summary of all 1136 proteins is shown in Table S2.

**Figure 4:**
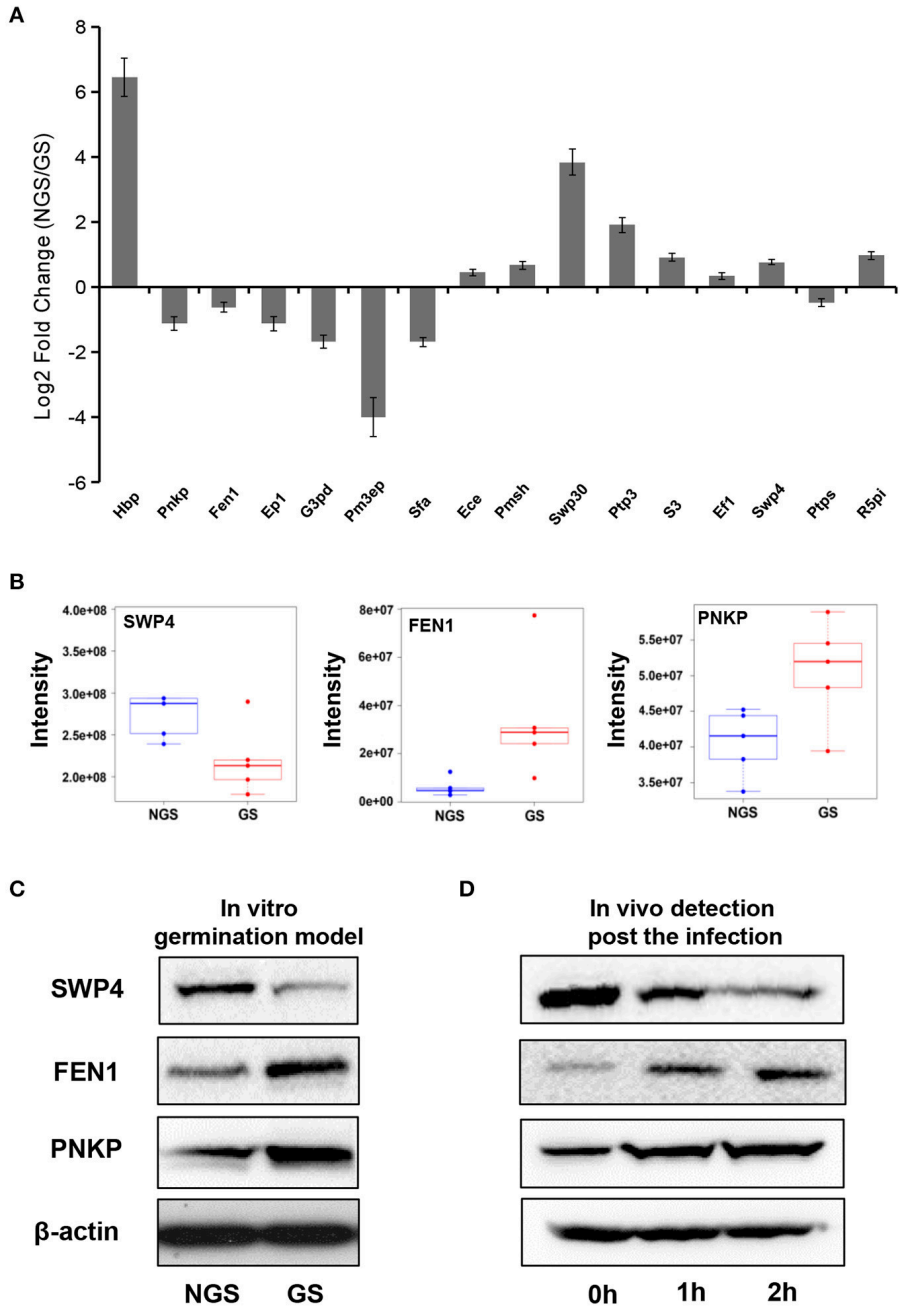
**Validation of significantly changed proteins by quantitative RT-PCR and Western blotting. (A)** The fold change of 16 randomly selected genes in the two groups (NGS and GS) measured by quantitative RT-PCR. The internal control is β-tubulin. Hbp, histone-binding protein RBBP4; Pnkp, bifunctional polynucleotide phosphatase/kinase; Fen1, flap endonuclease 1-A; Ep1, Exportin-1; G3pd, glyceraldehyde-3-phosphate dehydrogenase 2; Pm3ep, Pre-mRNA 3-end-processing factor FIP1; Sfa, splicing factor; Ece, exosome complex exonuclease RRP40; Pmsh, PRE-mRNA splicing helicase; Swp30, spore wall protein 30; Ptp3, polar tube protein 3; S3, septin 3; Ef1, elongation factor 1-alpha; Swp4, spore wall protein 4; Ptps, protein transport protein SEC23; R5pi, ribose-5-phosphate isomerase A. The fold change for each gene was determined from biological triplicates and were presented as the mean ± *SD*. **(B)** Comparison of the signal intensities of 3 selected differentially (*P* < 0.01) expressed proteins (SWP4, FEN1, and PNKP) obtained from the LFQ proteomic analysis. **(C)** Western blot analysis was used for validation of abundance changes for the 3 selected differentially expressed proteins during *N. bombycis* spore germination *in vitro*. **(D)** *In vivo* validation of 3 proteins of interest inside the gut of *B. mori* at different time points post infection. β-actin was used as control.

To investigate the exact protein content changes in *N. bombycis*, we performed a quantitative proteomic comparison between NGS and GS to better understand the important biological processes involved in this developmental stage. Using a *t*-test (*p* ≤ 0.05) as the variance for an up- or down-shift in abundance, the normalized ratio of protein abundance changes revealed significant and dynamic changes in protein profiles. Following this criteria of selection, 127 proteins were identified as significantly changed, with 60 proteins being up-regulated, and 67 proteins being down-regulated during the germination process. Volcano plot of all the identified proteins from NGS and GS is shown in Figure 3C. The molecular weight (MW), isoelectric point (PI), and grand average of hydropathicity (GRAVY) of the 127 significantly changed proteins were also investigated (Table S3). The top 20 significantly changed proteins with *p* ≤ 0.05 were listed in Table 1. The significantly up-regulated proteins were mainly infection-associated proteins (such as bifunctional polynucleotide phosphatase/kinase), spore proliferation-related proteins (transcriptional activator, Flap endonuclease 1-A, splicing factor and so on), spore wall proteins (putative SWP9), energy metabolism-related proteins (glyceraldehyde-3-phosphate dehydrogenase 2), proteasome-associated proteins (proteasome subunit alpha type-7, Proteasome component Y13), and so on. In contrast, the significantly down-regulated proteins mainly belonged to ribosomal proteins, SWP30 and putative SWP4, polar tube proteins, histone-binding proteins and so on (Table S3). To further validate the results from LFQ proteomic analysis, a set of 16 significantly changed proteins were selected randomly for quantitative RT-PCR analysis (Figure 4A). The expression patterns for all of these genes were consistent to the changes observed in related proteomic data. Scatter plots for LFQ intensity of 3 interesting proteins including SWP4, flap endonucleases 1 (FEN1) and polynucleotide kinase/phosphatase (PNKP; *P* < 0.01) were shown in Figure 4B. In addition, Western blotting also confirmed that two selected candidates, FEN1, and PNKP, were highly expressed in the GS group, while expression of SWP4 was higher in NGS (Figure 4C). Most importantly, FEN1 and PNKP were also highly expressed inside the gut of silkworms post infection, in contrast SWP4 was down regulated (Figure 4D).

**Table 1:** **Summary of the top 20 significantly changed proteins with *p* ≤ 0.05 (NGS/GS)**.

**GI number**	**Protein annotation**	**Ratio (NGS/GS)**	***p*-value (NGS/GS)**	**MW (KDa)**	**PI**	**Gravy**
gi|484852265	Protein transport protein SEC23	0.55	2.96E-08	16.25	9.35	−0.37
gi|484856373	Histone-binding protein RBBP4	2.77	8.59E-06	43.77	5.05	−0.37
gi|326559142	40S ribosomal protein S8-B	2.15	1.63E-05	19.00	10.27	−0.85
gi|484857162	Hypothetical protein NBO_12g0016	2.27	2.37E-05	40.10	7.99	−1.11
gi|484857067	Protein kinase domain Protein containing protein	0.75	6.79E-05	42.18	9.16	−1.08
gi|484856004	GPN-loop GTPase 1	0.16	7.34E-05	13.85	4.81	−0.15
gi|484854525	Hypothetical protein NBO_376g0001	2.18	8.47E-05	37.00	8.96	−0.6
gi|484857570	Hypothetical protein NBO_6gi003	2.5	1.24E-04	42.36	6.35	−0.87
gi|484856689	Hypothetical protein NBO_27g0020	2.28	1.78E-04	20.10	5.58	−0.65
gi|326565308	40S ribosomal protein S9	1.14	2.09E-04	21.56	9.99	−0.73
gi|326578181	60S ribosomal protein L36e	1.54	2.36E-04	11.12	10.72	−0.81
gi|326559144	40S ribosomal protein S8-B	8.51	3.63E-04	19.03	10.27	−0.83
gi|484856077	Transcriptional activator	0.23	4.96E-04	32.82	9.87	−1.28
gi|326578117	60S ribosomal protein L24	1.59	5.52E-04	10.28	11.09	−0.61
gi|326578097	60S ribosomal protein L4	1.36	5.67E-04	37.84	9.74	−0.4
gi|484856380	Glyceraldehyde-3-phosphate dehydrogenase 2, partial	0.83	6.21E-04	33.38	6.26	−0.29
gi|484854054	hypothetical protein NBO_462g0009	2.95	9.17E-04	18.37	7.84	−0.85
gi|259511816	Spore wall protein 30	1.71	9.24E-04	32.09	8.11	−0.1
gi|484856153	Hypothetical protein NBO_48g0007	0.42	9.40E-04	34.81	5.42	−0.73
gi|484853834	RING finger protein 121	0.48	9.42E-04	39.97	8.35	0.25

Altogether the quantitative RT-PCR and western blot analyses confirmed the results of the quantitative proteomic analysis, which revealed important players during the spore germination of *N. bombycis*.

### Functional categorization and enzyme code analysis

**Figure 5:**
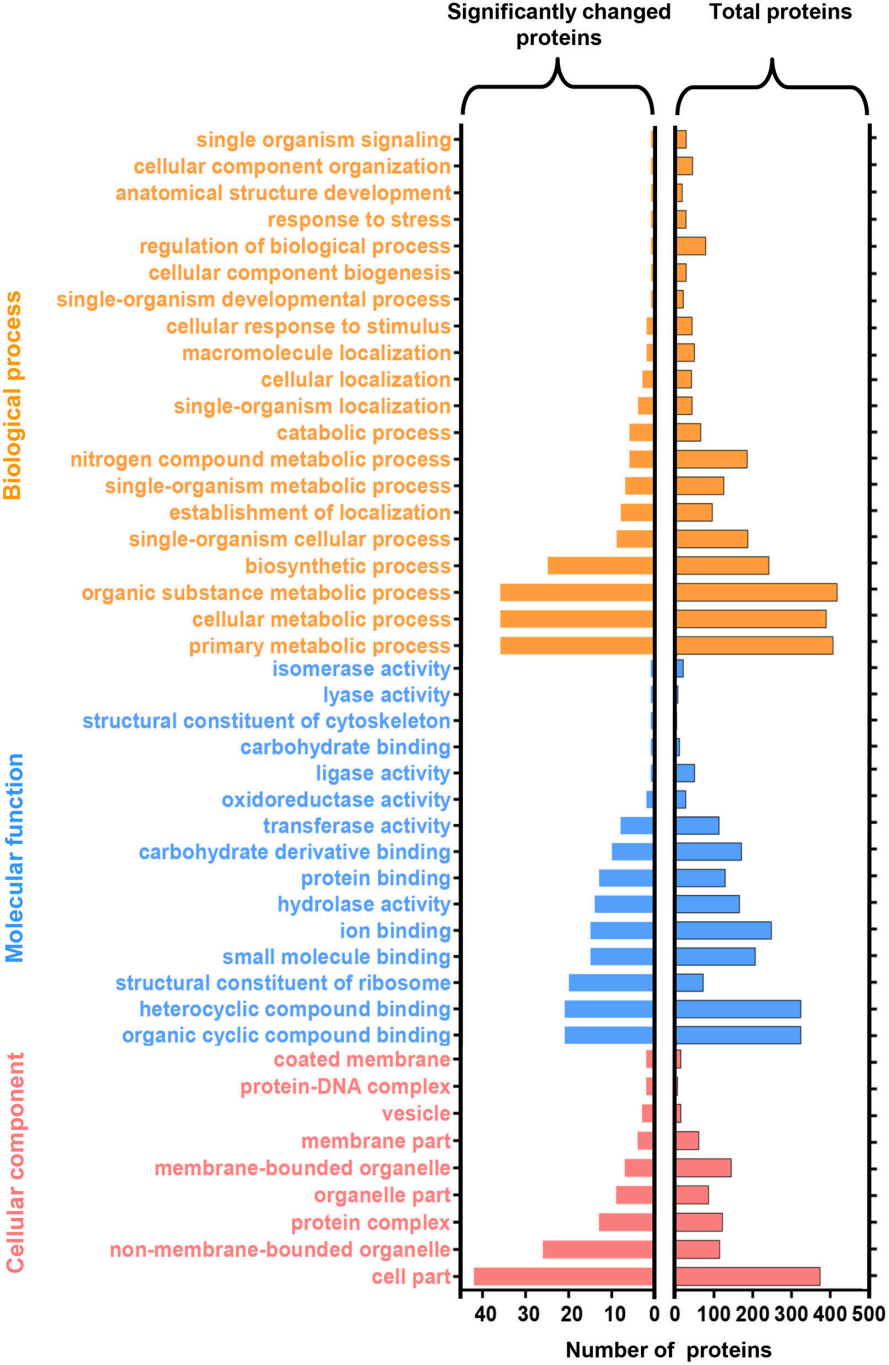
**Functional categorization of identified proteins upon *N. bombycis* spore germination**. A total of 1136 proteins were classified into 44 functional categories by Gene Ontology (GO) annotation (right panel). GO analysis of the 127 significantly changed proteins was showed in the left. GO terms are colored at level 2.

The identified protein sequences were assigned to Gene Ontology (GO) terms for functional classification. Three main categories of GO classification including biological process, molecular function, and cellular component, were analyzed separately to obtain the overall functional distribution of the identified proteins from NGS and GS. The resulting GO distributions for all 1136 proteins and for the 127 significantly changed proteins during germination were described in Figure 5, respectively. For all identified proteins, the most representative GO terms were organic substance metabolic process (GO:0071704), organic cyclic compound binding (GO:0097159), heterocyclic compound binding (GO:1901363), and cell part (GO:0044464). Whereas, the most representative GO terms for the 127 significantly changed proteins included: primary metabolic process (GO:0044238), cellular metabolic process (GO:0044237), and organic substance metabolic process (GO:0071704) within biological process; organic cyclic compound binding (GO:0097159), and heterocyclic compound binding (GO:1901363) within molecular function; and cell part (GO:0044464) within cellular component. Taken together, most of the significantly changed proteins were assigned to primary metabolic process, cellular metabolic process, and organic substance metabolic process, which indicates that the metabolic activity changed significantly after spore germination. Besides this, two proteins (transcriptional activator and transcription initiation factor) were identified in the transport (GO:0006810) and molecular function (GO:0003674) categories. Additionally, several ribosomal proteins were identified in the translation (GO:0006412) category, suggesting that the transcriptional and translation activities should be regulated after spore germination.

**Figure 6:**
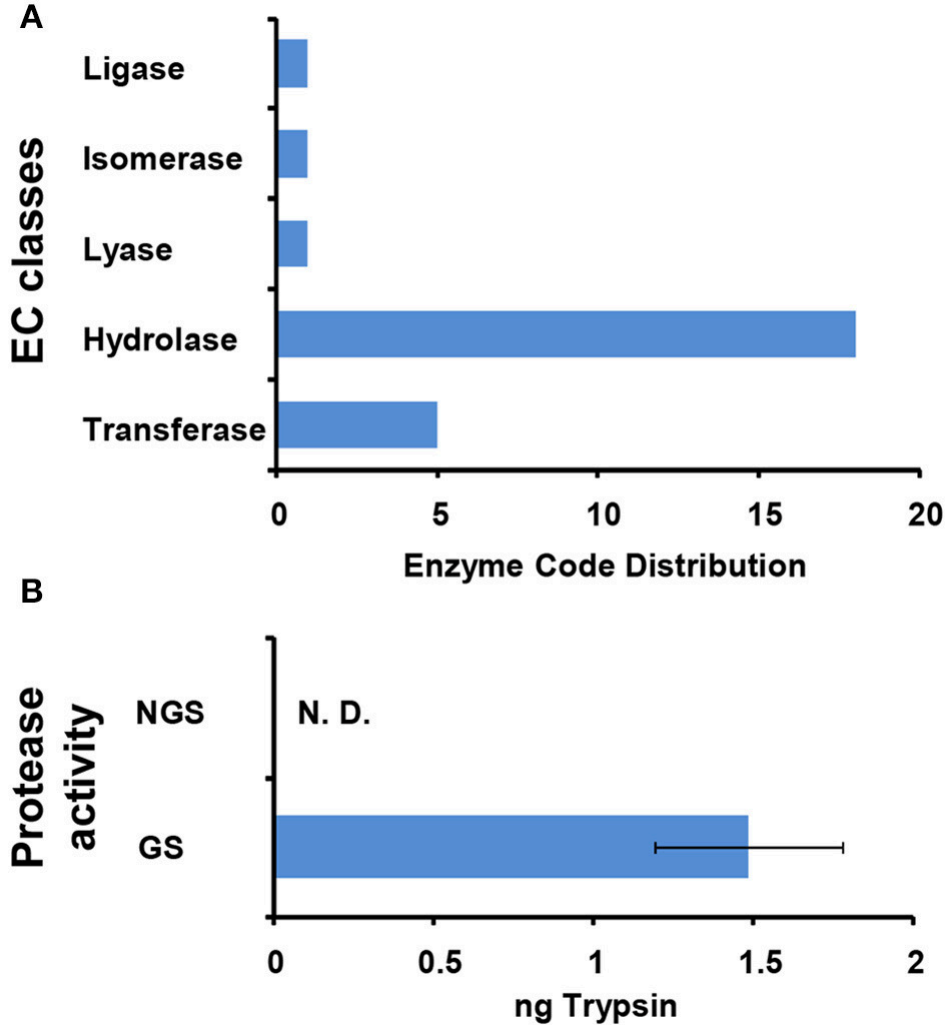
**Characterization of enzymes functioning in *N. bombycis* spore germination. (A)** Enzyme classification (EC) codes for the significantly changed proteins upon *N. bombycis* spore germination. Note that one sequence can be associated with more than one EC term. **(B)** The protease activities of GS supernatant and NGS supernatant. The absorbance of the dye-labeled peptide was measured at 570 nm for determination of the protease activity, equivalent to that of trypsin. Triplicate measurements were performed. Data are shown as the mean ± *SD*. GS supernatant showed proteolytic activity. N. D., not detected.

The 127 significantly changed proteins were further analyzed with enzyme codes (Figure 6A). Totally, 18 enzyme proteins were identified including 11 hydrolases, four transferases, one lyase, one isomerase, and one ligase. Interestingly, according to the LFQ analysis, most of enzyme proteins including nine hydrolases, three transferases, one lyase, and one ligase were significantly up-regulated after *N. bombycis* spore germination. For hydrolases, we paid attention to five proteinases and cleavage and polyadenylation specificity factor (CPSF) subunit 3. The proteasome degrades unneeded or damaged proteins by proteolysis to control protein stability in the organism. CPSF is involved in the cleavage of the 30027-signaling region from a newly synthesized pre-messenger RNA (pre-mRNA) molecule during gene transcription. Leucyl tRNA synthetase, which promotes the ligation of leucine to tRNA, was significantly up-regulated too after spore germination (Table S3). We further compared the specific activity of enzymes important for protein degradation from supernatants of GS and NGS. GS exhibited significant protease activity (Figure 6B).

### Metabolic pathway analysis mapping the identified proteins to KEGG pathways

**Figure 7:**
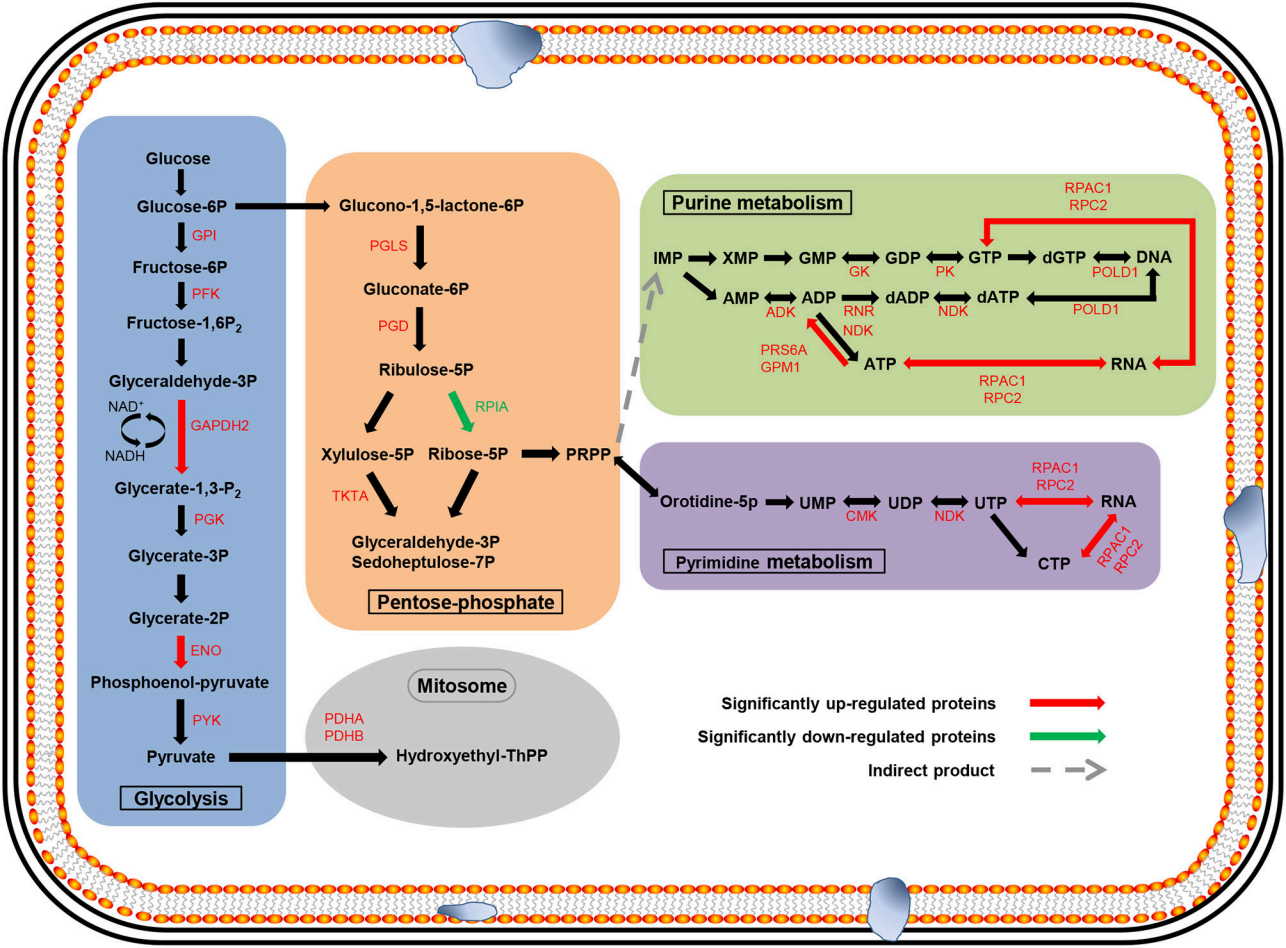
**Major metabolic pathways anticipated to participate in spore germination process in *N. bombycis***. The proteomic data was projected to the KEGG pathway database. Significantly changed pathways involve: glycolysis (blue), pentose-phosphate (orange), purine metabolism (green), pyrimidine metabolism (purple), and metabolism in mitosome (gray). The metabolites are in black. The up-regulated enzymes are highlighted in red and down-regulated proteins in green. Red and green arrows represent significantly changed proteins (*p* < 0.05).

To identify the related biological pathways that are active during *N. bombycis* spore germination, all the 1136 identified proteins were searched against pathway collections in the KEGG database. All proteins were mapped into 37 metabolic pathways (Table S4). And the enrichment of the 127 significantly changed proteins in each pathway was obtained by calculating the probability using hypergeometric distribution. As shown in Table S4, most identified proteins were mapped to the purine metabolism and the number of mapped proteins was 96. Another 70 identified proteins were mapped to the thiamine metabolism. For the 127 significantly changed proteins, nine pathways (including Glycolysis/Gluconeogenesis, Pentose phosphate pathway, Purine metabolism, Pyrimidine metabolism, Biosynthesis of antibiotics, Fructose and mannose metabolism, Thiamine metabolism) were enriched (Table S5 and Dataset S6). Glycolysis/Gluconeogenesis and pentose phosphate pathways should participate in the energy metabolism process during spore germination and some key relevant proteins were identified (Figure 7). Almost all of the enzymes involved in glycolysis and pentose phosphate pathway (except ribulose-5P epimerase and hexokinase) and two subunits from the downstream pyruvate dehydrogenase enzyme were observed, with the majority of them showing an up-regulation. More importantly, three of these enzymes showed significantly differential regulation (*P* < 0.05) in protein expression levels after germination (Figure 7). Six enzymes were mapped to the purine metabolism, which are the most abundant enzymes for any of the pathways. Two enzymes were also mapped to the pyrimidine metabolism (Table 2). The two pathways are important for nucleotide synthesis.

**Table 2:** **The significantly changed proteins involved in the purine and pyrimidine metabolism**.

**Pathway**	**Proteins in pathway**	**Enzyme**	**GI number**	**Protein annotation**	**Ratio (NGS/GS)**	***p*-value (NGS/GS)**
Purine metabolism	6	Phosphatase	gi|484855499	26S protease regulatory subunit 6A	0.69	2.99E-03
			gi|484856004	GPN-loop GTPase 1	0.16	7.34E-05
			gi|484855718	PRE-mRNA splicing helicase	2.22	7.20E-03
			gi|484856166	Tubulin alpha chain	1.29	4.41E-03
		Adenylpyro-phosphatase	gi|484855499	26S protease regulatory subunit 6A	0.69	2.99E-03
		RNA polymerase	gi|484853415	DNA-directed RNA polymerases I and III subunit RPAC1	0.44	1.18E-02
			gi|484854029	DNA-directed RNA polymerase III subunit RPC2	0.22	1.81E-02
Pyrimidine metabolism	2	RNA polymerase	gi|484853415	DNA-directed RNA polymerases I and III subunit RPAC1	0.44	1.18E-02
			gi|484854029	DNA-directed RNA polymerase III subunit RPC2	0.22	1.81E-02

## Discussion

As a turning point from the dormant state to the infected state and the first step in host cell invasion, spore germination is believed to be crucial for the success of microsporidian pathogens. A better understanding of the germination process therefore could make important contributions to the studies on this specific mode of infection and to the development of biological controlling strategies of these economically important and human-related pathogens. The entire protein coding sequences annotated in the *N. bombycis* genome is 4468 (Pan et al., 2013), and 1136 proteins were identified and quantified in our proteomic analysis of *N. bombycis* spores. In contrast, only 141 of the total 2573 predicted proteins were obtained in a spore germination study of the microsporidia *Spraguea lophii* using complex-mix proteomics (Campbell et al., 2013). Therefore, the LFQ proteomic approach employed in our study was sufficient for the microsporidia proteomic study.

Some major structural proteins involved in host-pathogen interactions have been characterized in the current work. We found that polar tube proteins (PTPs) 1 and 3 were significantly down-regulated during *N. bombycis* spore germination. It has been shown in another microsporidian *Encephalitozoon cuniculi* that PTPs accumulate in the sporogony stage but play roles in the germination process (Grisdale et al., 2013). Spore wall proteins, such as SWP26, SWP30, and SWP32, changed significantly too. It is known that SWP30 could not interact with the spore coat after alkali treatment (Yang et al., 2014). The *N. bombycis* germination process is generally triggered by alkali conditions inside the intestine of silkworms. Therefore, the decreased SWP30 level observed in our study is consistent with previous results. Similarly, SWP4 also showed a significant down-regulation, but its biological function has not been revealed until now. These proteins probably play important roles in the spore germination process and deserve further studies. A recent report showed that there was an interaction between SWP7 and SWP9: SWP9 in the chitin layer of the spore wall could act as a scaffolding protein that supports SWP7 and the two proteins were confirmed to mediate the infectious process by enabling spore adherence to host cells (Yang et al., 2015). Both SWP9 and SWP7 were confirmed to accumulate after germination at the proteomics level in our study, which might promote adherence to host cells. Currently many detection methods for *N. bombycis* SWPs have been established, such as monoclonal antibody (Li et al., 2015), and electrochemical immunosensor (Wang et al., 2014). The enrichment and purification methods of *N. bombycis* SWPs were also optimized (Yang et al., 2014; Zhao W. et al., 2015). However, the pertinence of detection is limited due to multiple types of SWPs. Spore germination is the important step for invasion. The significantly changed proteins obtained in this study might be related with spore germination and invasion. According to the analysis of SWPs here, the pertinence and efficiency of detection could be enhanced.

Other interesting proteins that showed an significant change in our study included a polynucleotide kinase/phosphatase (PNKP) and a RNA ligase 1 (RNL 1), both of which are the prototypal RNA healing and sealing enzymes involved in the RNA repair pathway (Novogrodsky et al., 1966; Novogrodsky and Hurwitz, 1966; Cameron and Uhlenbeck, 1977; Sugino et al., 1977). It is reported in phage that PNKP provides phages a way to evade RNA-damaging antiviral host response (Zhu et al., 2004). The increase of PNKP indicates that it might help *N. bombycis* evade the RNA-damaging mediated by host immune response and contribute to the normal growth of the pathogen inside. Flap endonucleases 1 (FEN1), found in diverse organisms, is a key nuclease that plays a role in DNA replication, repair, and recombination (Sayers, 1994; Ceska and Sayers, 1998; Liu et al., 2004). FEN1 was identified as a significantly up-regulated protein too upon spore germination. This increase might be related to the replication of double-stranded DNA during the pathogen proliferative phase. FEN1 could also take part in DNA repair pathway via the base excision repair pathway (Dianov and Lindahl, 1994), suggesting that it probably also protects *N. bombycis* from DNA-damage. These new findings provide additional perspectives for the complex *B. mori*-*N. bombycis* interactions.

According to the enzyme classification of LFQ data, 18 enzymes including five proteinases and the CPSF subunit 3 were found to be significantly up-regulated after spore germination. The protease activity was confirmed in the supernatant of GS. These released proteases may participate in the digestion of the host cell or the resistance of the host immune defense, and then make contributions to the spore infection and proliferation. As an important functional protein, the proteasome could play a role in cell cycle control (Chesnel et al., 2006), apoptosis (Haas et al., 1995; He et al., 2015), response to cellular stress (Garrido et al., 2006), and immunoreaction (Wang and Maldonado, 2006). CPSF is an important protein which could play a role in mRNA transcription. The significant up-regulations of these proteins indicate the levels of protein regulation are enhanced during the germination process. Furthermore, based on the significant up-regulation of leucyl tRNA synthetase, we inferred that there were strong synthetic demands for leucyl-related proteins in the spore germination. As a metabolic substrate, leucyl takes part in protein synthesis and could regulate and control gene transcription, translation, and post-translational modifications. Up to now, the role of leucyl in spore germination has been scarcely reported.

It is well-accepted that the microsporidian proliferative phase begins along with the activation of pathways related to energy generation and substance synthesis after spore germination. Comparative genomics research revealed that *N. bombycis* lacked genes that are required for oxidative phosphorylation, the tricarboxylic acid cycle, and fatty acid β-oxidation (Pan et al., 2013). Glycolysis, the pentose phosphate pathway, and a trehalose metabolism were thought to be the backbone for microsporidian energy metabolism because their mitochondria are massively reduced (Williams and Keeling, 2003; Van der Giezen et al., 2005). Mitosomes, which are a residual mitochondrion-derived organelle, have been regarded as the main organelle involved in energy metabolism in *Encephalitozoon cuniculi* (Katinka et al., 2001). In our study, almost all of the enzymes involved in glycolysis and the pentose phosphate pathway and two downstream pyruvate dehydrogenase enzymes were identified, furthermore three of these enzymes showed significantly differential regulation in protein expression levels after germination. Analysis of the two pathways provided the evidence to better understand the mode of energy generation in *N. bombycis*. We also identified 15 significantly up-regulated proteins involved in DNA transcription, translation, and transport after GO classification (Table S3). In contrast, histone-binding protein (RBBP4) was significantly down-regulated, which was connected to transcriptional silencing (Wolffe et al., 2000). With the help of the KEGG pathway analysis, most of the enzymes were mapped to the purine metabolism and two enzymes to the pyrimidine metabolism (Table 2). Nucleotide metabolism, which supports the DNA and RNA synthesis, is the very important metabolic pathway in free-living bacteria, Archaea, as well as microsporidia (Kanehisa et al., 2002). The nucleotides participate in a wide range of cellular functions, such as energy transduction, biomolecules syntheses, and signaling pathway (Welin and Nordlund, 2010). Purine and pyrimidine can be provided from *de novo* biosynthetic pathways or supplied via salvage pathways. Some microsporidia acquired genes involved in nucleic acid synthesis by horizontal gene transfer. The utilization of nucleotides and adaptability in host of microsporidia were enhanced by these genes (Alexander et al., 2016). In addition, some enzymes for the transformation of purine and pyrimidine during different phosphorylation and oxidation states were identified in microsporidia. These enzymes play key roles in the nucleotide metabolism too and help to better use of nucleotides for microsporidia (Heinz et al., 2014). Altogether, these results indicate that metabolic activities of *N. bombycis* are significantly enhanced during spore germination, which provide striking support for the following pathogen invasion and proliferation inside the host.

The spore germination is an intricate and elaborate process. Our study presents the first global quantitative proteomic analysis of *N. bombycis* spores during the germination process. Despite the proteome is considerably complex, a set of proteins related to cytoarchitecture, energy metabolism, and host immunity resistance have been identified as key players upon spore germination. Not only help to gain a better understanding of the microsporidia germination, this research is also a stepping stone on the way to further study of unique infection mode of this economically important pathogen and other microsporidia in general.

## Author contributions

YS and XLu contributed to the initial design of the research. HL conducted the experiment and BC performed bioinformatics analyses with guidance from YS. SH and XLi collected samples. YS, XLu, and HL prepared the draft of this publication and all authors contributed to the subsequent stages of manuscript preparation.

### Conflict of interest statement

The authors declare that the research was conducted in the absence of any commercial or financial relationships that could be construed as a potential conflict of interest.

## Supplementary material

Table S1 **PCR primers used for the qRT-PCR validation**.

Table S2 **Summary of the 1136 identified proteins in NGS and GS using LFQ proteomic analysis**.

Table S3 **Summary of the 127 significantly changed proteins with *p* ≤ 0.05 (NGS/GS)**.

Table S4 **KEGG mapping analysis for the 1136 proteins between NGS and GS**.

Table S5 **KEGG mapping analysis for the 127 significantly changed proteins between NGS and GS**.

Dataset S6 **KEGG pathway diagrams for the 127 significantly changed proteins**.

## References

[B1] Alexander W. G. Wisecaver J. H. Rokas A. Hittinger C. T. (2016). Horizontally acquired genes in early-diverging pathogenic fungi enable the use of host nucleosides and nucleotides. Proc. Natl. Acad. Sci. U.S.A. 113, 4116–4121. 10.1073/pnas.1517242113 27035945 PMC4839431

[B2] Becnel J. J. Andreadis T. G. (1999). Microsporidia in insect, in The Microsporidia and Microsporidiosis, ed Wittner M. Weiss L. M. (Washington, DC: ASM Press), 447–501.

[B3] Bhat S. A. Bashir I. Kamili A. S. (2009). Microsporidiosis of silkworm, *Bombyx mori* L. (Lepidoptera- bombycidae): a review. Afr. J. Agr. Res. 4, 1519–1523.

[B4] Cai S. Lu X. Qiu H. Li M. Feng Z. (2011). Identification of a *Nosema bombycis* (Microsporidia) spore wall protein corresponding to spore phagocytosis. Parasitology 138, 1102–1109. 10.1017/S0031182011000801 21756420

[B5] Cameron V. Uhlenbeck O. C. (1977). 3′-Phosphatase activity in T4 polynucleotide kinase. Biochemistry 16, 5120–5126. 10.1021/bi00642a027 199248

[B6] Campbell S. E. Williams T. A. Yousuf A. Soanes D. M. Paszkiewicz K. H. Williams B. A. P. (2013). The genome of *Spraguea lophii* and the basis of host-microsporidian interactions. PLoS Genet. 9:e1003676. 10.1371/journal.pgen.1003676 23990793 PMC3749934

[B7] Ceska T. A. Sayers J. R. (1998). Structure-specific DNA cleavage by 5′ nucleases. Trends Biochem. Sci. 23, 331–336. 10.1016/S0968-0004(98)01259-6 9787638

[B8] Chesnel F. Bazile F. Pascal A. Kubiak J. Z. (2006). Cyclin B dissociation from CDK1 precedes its degradation upon MPF inactivation in mitotic extracts of *Xenopus laevis* embryos. Cell Cycle 5, 1687–1698. 10.4161/cc.5.15.3123 16921258

[B9] Conesa A. Gotz S. Garcia-Gomez J. M. Terol J. Talon M. Robles M. (2005). Blast2GO: a universal tool for annotation, visualization and analysis in functional genomics research. Bioinformatics 21, 3674–3676. 10.1093/bioinformatics/bti610 16081474

[B10] Cox J. Matic I. Hilger M. Nagaraj N. Selbach M. Olsen J. V.. (2009). A practical guide to the MaxQuant computational platform for SILAC-based quantitative proteomics. Nat. Protoc. 4, 698–705. 10.1038/nprot.2009.36 19373234

[B11] Dianov G. Lindahl T. (1994). Reconstitution of the DNA base excision-repair pathway. Curr. Biol. 4, 1069–1076. 10.1016/S0960-9822(00)00245-1 7535646

[B12] Dow J. A. (1992). pH GRADIENTS IN LEPIDOPTERAN MIDGUT. J. Exp. Biol. 172, 355–375. 9874748 10.1242/jeb.172.1.355

[B13] Garrido C. Brunet M. Didelot C. Zermati Y. Schmitt E. Kroemer G. (2006). Heat shock proteins 27 and 70: anti-apoptotic proteins with tumorigenic properties. Cell Cycle 5, 2592–2601. 10.4161/cc.5.22.3448 17106261

[B14] Gotz S. Garcia-Gomez J. M. Terol J. Williams T. D. Nagaraj S. H. Nueda M. J.. (2008). High-throughput functional annotation and data mining with the Blast2GO suite. Nucleic Acids Res. 36, 3420–3435. 10.1093/nar/gkn176 18445632 PMC2425479

[B15] Grisdale C. J. Bowers L. C. Didier E. S. Fast N. M. (2013). Transcriptome analysis of the parasite *Encephalitozoon cuniculi*: an in-depth examination of pre-mRNA splicing in a reduced eukaryote. BMC Genomics 14:207. 10.1186/1471-2164-14-207 23537046 PMC3629993

[B16] Haas A. L. Baboshina O. Williams B. Schwartz L. M. (1995). Coordinated induction of the ubiquitin conjugation pathway accompanies the developmentally programmed death of insect skeletal muscle. J. Biol. Chem. 270, 9407–9412. 10.1074/jbc.270.16.9407 7721865

[B17] He X. Fu Z. Li M. Liu H. Cai S. Man N.. (2015). *Nosema bombycis* (Microsporidia) suppresses apoptosis in *Bm*N cells (Bombyx mori). Acta Biochim. Biophys. Sin. (Shanghai) 47, 696–702. 10.1093/abbs/gmv062 26188202

[B18] Heinz E. Hacker C. Dean P. Mifsud J. Goldberg A. V. Williams T. A.. (2014). Plasma membrane-located purine nucleotide transport proteins are key components for host exploitation by microsporidian intracellular parasites. PLoS Pathog. 10:e1004547. 10.1371/journal.ppat.1004547 25474405 PMC4256464

[B19] Jia B. Liu J. Van Duyet L. Sun Y. Xuan Y. H. Cheong G. W. (2015). Proteome profiling of heat, oxidative, and salt stress responses in *Thermococcus kodakarensis* KOD1. Front. Microbiol. 6:605. 10.3389/fmicb.2015.00605 26150806 PMC4473059

[B20] Kanehisa M. Goto S. Kawashima S. Nakaya A. (2002). The KEGG databases at GenomeNet. Nucleic Acids Res. 30, 42–46. 10.1093/nar/30.1.42 11752249 PMC99091

[B21] Katinka M. D. Duprat S. Cornillot E. Metenier G. Thomarat F. Prensier G.. (2001). Genome sequence and gene compaction of the eukaryote parasite *Encephalitozoon cuniculi*. Nature 414, 450–453. 10.1038/35106579 11719806

[B22] Li Y. Tao M. Ma F. Pan G. Zhou Z. Wu Z. (2015). A monoclonal antibody that tracks endospore formation in the microsporidium *Nosema bombycis*. PLoS ONE 10:e121884. 10.1371/journal.pone.0121884 25811182 PMC4374874

[B23] Li Y. Wu Z. Pan G. He W. Zhang R. Hu J.. (2009). Identification of a novel spore wall protein (SWP26) from microsporidia *Nosema bombycis*. Int. J. Parasitol. 39, 391–398. 10.1016/j.ijpara.2008.08.011 18854188

[B24] Li Z. Pan G. Li T. Huang W. Chen J. Geng L.. (2012). SWP5, a spore wall protein, interacts with polar tube proteins in the parasitic microsporidian *Nosema bombycis*. Eukaryotic Cell 11, 229–237. 10.1128/EC.05127-11 22140229 PMC3272902

[B25] Liu Y. Kao H. I. Bambara R. A. (2004). Flap endonuclease 1: a central component of DNA metabolism. Annu. Rev. Biochem. 73, 589–615. 10.1146/annurev.biochem.73.012803.092453 15189154

[B26] Mei L. L. Jin W. (1989). Studies on *Nosema bombycis* and *Nosema hemerophila*. Sci Seric. 15, 135–138.

[B27] Novogrodsky A. Hurwitz J. (1966). The enzymatic phosphorylation of ribonucleic acid and deoxyribonucleic acid. I. Phosphorylation at 5′-hydroxyl termini. J. Biol. Chem. 241, 2923–2932. 4287929

[B28] Novogrodsky A. Tal M. Traub A. Hurwitz J. (1966). The enzymatic phosphorylation of ribonucleic acid and deoxyribonucleic acid. II. Further properties of the 5′-hydroxyl polynucleotide kinase. J. Biol. Chem. 241, 2933–2943. 4287930

[B29] Pan G. Xu J. Li T. Xia Q. Liu S. Zhang G.. (2013). Comparative genomics of parasitic silkworm microsporidia reveal an association between genome expansion and host adaptation. BMC Genomics 14:186. 10.1186/1471-2164-14-186 23496955 PMC3614468

[B30] Sayers J. R. (1994). Computer aided identification of a potential 5′-3′ exonuclease gene encoded by *Escherichia coli*. J. Theor. Biol. 170, 415–421. 10.1006/jtbi.1994.1202 7996866

[B31] Schmittgen T. D. Livak K. J. (2008). Analyzing real-time PCR data by the comparative C(T) method. Nat. Protoc. 3, 1101–1108. 10.1038/nprot.2008.73 18546601

[B32] Schottelius J. Schmetz C. Kock N. P. Schuler T. Sobottka I. Fleischer B. (2000). Presentation by scanning electron microscopy of the life cycle of microsporidia of the genus *Encephalitozoon*. Microbes Infect. 2, 1401–1406. 10.1016/S1286-4579(00)01293-4 11099925

[B33] Sudakaran S. Salem H. Kost C. Kaltenpoth M. (2012). Geographical and ecological stability of the symbiotic mid-gut microbiota in European firebugs, *Pyrrhocoris apterus* (Hemiptera, Pyrrhocoridae). Mol. Ecol. 21, 6134–6151. 10.1111/mec.12027 23017151

[B34] Sugino A. Snoper T. J. Cozzarelli N. R. (1977). Bacteriophage T4 RNA ligase. Reaction intermediates and interaction of substrates. J. Biol. Chem. 252, 1732–1738. 320212

[B35] Tao Y. Fang L. Yang Y. Jiang H. Yang H. Zhang H.. (2013). Quantitative proteomic analysis reveals the neuroprotective effects of huperzine A for amyloid beta treated neuroblastoma N2a cells. Proteomics 13, 1314–1324. 10.1002/pmic.201200437 23424162

[B36] Troemel E. R. Becnel J. J. (2015). Genome analysis and polar tube firing dynamics of mosquito-infecting microsporidia. Fungal Genet. Biol. 83, 41–44. 10.1016/j.fgb.2015.08.007 26300319 PMC4587305

[B37] Van der Giezen M. Tovar J. Clark C. G. (2005). Mitochondrion-derived organelles in protists and fungi. Int. Rev. Cytol. 244, 175–225. 10.1016/S0074-7696(05)44005-X 16157181

[B38] Vavra J. Larsson J. I. R. (1999). Structure of the microsporidia, in The Microsporidia and Microsporidiosis, eds Wittner M. Weiss L. M. (Washington, DC: ASM Press), 7–84.

[B39] Vavra J. Lukes J. (2013). Microsporidia and ‘the art of living together’. Adv. Parasitol. 82, 253–319. 10.1016/B978-0-12-407706-5.00004-6 23548087

[B40] Wang J. Maldonado M. A. (2006). The ubiquitin-proteasome system and its role in inflammatory and autoimmune diseases. Cell. Mol. Immunol. 3, 255–261. 16978533

[B41] Wang Q. Song Y. Chai Y. Pan G. Li T. Yuan Y.. (2014). Electrochemical immunosensor for detecting the spore wall protein of *Nosema bombycis* based on the amplification of hemin/G-quadruplex DNAzyme concatamers functionalized Pt@Pd nanowires. Biosens. Bioelectron. 60, 118–123. 10.1016/j.bios.2014.03.075 24787126

[B42] Wang Y. Dang X. Ma Q. Liu F. Pan G. Li T.. (2015). Characterization of a novel spore wall protein *Nb*SWP16 with proline-rich tandem repeats from *Nosema bombycis* (microsporidia). Parasitology 142, 534–542. 10.1017/S0031182014001565 25363531

[B43] Welin M. Nordlund P. (2010). Understanding specificity in metabolic pathways–structural biology of human nucleotide metabolism. Biochem. Biophys. Res. Commun. 396, 157–163. 10.1016/j.bbrc.2010.04.054 20494131

[B44] Williams B. A. Keeling P. J. (2003). Cryptic organelles in parasitic protists and fungi. Adv. Parasitol. 54, 9–68. 10.1016/S0065-308X(03)54001-5 14711083

[B45] Wisniewski J. R. Nagaraj N. Zougman A. Gnad F. Mann M. (2010). Brain phosphoproteome obtained by a FASP-based method reveals plasma membrane protein topology. J. Proteome Res. 9, 3280–3289. 10.1021/pr1002214 20415495

[B46] Wolffe A. P. Urnov F. D. Guschin D. (2000). Co-repressor complexes and remodelling chromatin for repression. Biochem. Soc. Trans. 28, 379–386. 10.1042/bst0280379 10961924

[B47] Yang D. Dang X. Tian R. Long M. Li C. Li T.. (2014). Development of an approach to analyze the interaction between *Nosema bombycis* (microsporidia) deproteinated chitin spore coats and spore wall proteins. J. Invertebr. Pathol. 115, 1–7. 10.1016/j.jip.2013.10.004 24161881

[B48] Yang D. Pan G. Dang X. Shi Y. Li C. Peng P.. (2015). Interaction and assembly of two novel proteins in the spore wall of the microsporidian species *Nosema bombycis* and their roles in adherence to and infection of host cells. Infect. Immun. 83, 1715–1731. 10.1128/IAI.03155-14 25605761 PMC4363453

[B49] Zhang D. Li S. Hu L. Sheng L. Chen L. (2015). Modulation of protease-activated receptor expression by *Porphyromonas gingivalis* in human gingival epithelial cells. BMC Oral Health 15:128. 10.1186/s12903-015-0105-8 26476532 PMC4609475

[B50] Zhang F. Lu X. Kumar V. S. Zhu H. Chen H. Chen Z.. (2007). Effects of a novel anti-exospore monoclonal antibody on microsporidial *Nosema bombycis* germination and reproduction *in vitro*. Parasitology 134, 1551–1558. 10.1017/S0031182007002934 17577423

[B51] Zhao Q. Gao J. Suo J. Chen S. Wang T. Dai S. (2015). Cytological and proteomic analyses of horsetail (*Equisetum arvense* L.) spore germination. Front. Plant Sci. 6:441. 10.3389/fpls.2015.00441 26136760 PMC4469821

[B52] Zhao W. Hao Y. Wang L. Zhou Z. Li Z. (2015). Development of a strategy for the identification of surface proteins in the pathogenic microsporidian *Nosema bombycis*. Parasitology 142, 865–878. 10.1017/S0031182015000190 25811320

[B53] Zhu F. Shen Z. Hou J. Zhang J. Geng T. Tang X.. (2013). Identification of a protein interacting with the spore wall protein SWP26 of *Nosema bombycis* in a cultured BmN cell line of silkworm. Infect. Genet. Evol. 17, 38–45. 10.1016/j.meegid.2013.03.02 23542093

[B54] Zhu H. Yin S. Shuman S. (2004). Characterization of polynucleotide kinase/phosphatase enzymes from Mycobacteriophages omega and Cjw1 and vibriophage KVP40. J. Biol. Chem. 279, 26358–26369. 10.1074/jbc.M403200200 15056675

