# Supplementary material for: Quantitative Proteomic Analysis of Germination of Nosema bombycis Spores under Extremely Alkaline Conditions
Source: Front Microbiol. 2016 Sep 21;7:1459. doi: 10.3389/fmicb.2016.01459 (PMC5030232; doi:10.3389/fmicb.2016.01459)
Supplement: Table S1 — PCR primers used for the qRT-PCR validation. [file Table1.docx]

**S1 Table.** **PCR primers used for the** **qRT-PCR validation.**

| Proteins | Primer sequences |
| --- | --- |
| Histone-binding protein RBBP4  Bifunctional polynucleotide phosphatase/kinase  Flap endonuclease 1-A  Exportin-1  Glyceraldehyde-3-phosphate dehydrogenase 2, partial  Pre-mRNA 3-end-processing factor FIP1, partial  Splicing factor, arginine/serine-rich 10  Exosome complex exonuclease RRP40  PRE-mRNA splicing helicase  Spore wall protein 30  Polar tube protein 3  septin 3, partial  Elongation factor 1-alpha  hypothetical spore wall protein  Protein transport protein SEC23  Ribose-5-phosphate isomerase A  β-tubulin | F: 5’- aaacatactcttctatctttccctg-3’  R: 5’- tgtacttgcttcttcatcatcag-3’  F: 5’- ataacgccaggtttcaaagaggt-3’  R: 5’- ggcattcataaagaaagttaccaaa-3’  F: 5’- taacattatcatctgtacctaaagcag-3’  R: 5’- agaaaggaatcgtagaaagaccc-3’  F: 5’- aatcttcatcattcccgaactca-3’  R: 5’- ctcaaaacttccctaatatcaca-3’  F: 5’- atcaatggctttggaagaatcgg’  R: 5’- aagggagtttggctgtagaatgg-3’  F: 5’- ataaaccgggtgctgatgttacgg-3’  R: 5’- atcgcactgtctcctgttcctattctg-3’  F: 5’- ttcggtttgagtattcacgccact-3’  R: 5’- catttaccagccagcctttcctt-3’  F: 5’- ctctaagttttacgaatgcctct-3’  R: 5’- atcatcacaaacccatttagtcc-3’  F: 5’- tattgccagctaactaaaccgaaca-3’  R: 5’- attgccttgaatgcagtcggaag-3’  F: 5’- catcaaacttgtttctcgtcgtt-3’  R: 5’- cagaggcaatccttctatcatcc-3’  F: 5’- ctgctcacattgctatcgctcac-3’  R: 5’- taggcttctccaacattgcttacttct-3’  F: 5’- cttgttttactcctcagcctatt-3’  R: 5’- cctttctaaagcacttttcctaa-3’  F: 5’- agccaagaccaaagtaatagcag-3’  R: 5’- tgggacgtgagtaattccaagat-3’  F: 5’- tcttactaaatacgcccaaactg-3’  R: 5’- ataaacagagcaaatagcaccac-3’  F: 5’- gagtcgtatttatcacaaggttt-3’  R:5’- tttcctatcacttctttggtatt-3’  F: 5’- taatcccacaatcactttcttat-3’  R:5’- ccgtgttcaactaccccactttt-3’  F: 5’- GACTGTAGCTGCTGTCTTTA-3’  R: 5’- GCAGTAGTATTTCCCATAAA-3’ |
