# Supplementary material for: Quantitative Proteomic Analysis of Germination of Nosema bombycis Spores under Extremely Alkaline Conditions
Source: Front Microbiol. 2016 Sep 21;7:1459. doi: 10.3389/fmicb.2016.01459 (PMC5030232; doi:10.3389/fmicb.2016.01459)
Supplement: Table S3 — Summary of the 127 significantly changed proteins with p ≤ 0.05 (NGS/GS). [file Table3.docx]

**S3 Table. Summary of the 127 significantly changed proteins with *p*-value ≤0.05 (NGS/GS).**

| GI number | Protein annotation | Ratio (NGS/GS) | *p*-value (NGS/GS) | MW  (KDa) | PI | Gravy |
| --- | --- | --- | --- | --- | --- | --- |
| gi\|484852265 | Protein transport protein SEC23 | 0.55 | 2.96E-08 | 16.25 | 9.35 | -0.37 |
| gi\|484856373 | Histone-binding protein RBBP4 | 2.77 | 8.59E-06 | 43.77 | 5.05 | -0.37 |
| gi\|326559142 | 40S ribosomal protein S8-B | 2.15 | 1.63E-05 | 19.00 | 10.27 | -0.85 |
| gi\|484857162 | hypothetical protein NBO_12g0016 | 2.27 | 2.37E-05 | 40.10 | 7.99 | -1.11 |
| gi\|484857067 | protein kinase domain Protein containing protein | 0.75 | 6.79E-05 | 42.18 | 9.16 | -1.08 |
| gi\|484856004 | GPN-loop GTPase 1 | 0.16 | 7.34E-05 | 13.85 | 4.81 | -0.15 |
| gi\|484854525 | hypothetical protein NBO_376g0001 | 2.18 | 8.47E-05 | 37.00 | 8.96 | -0.60 |
| gi\|484857570 | hypothetical protein NBO_6gi003 | 2.50 | 1.24E-04 | 42.36 | 6.35 | -0.87 |
| gi\|484856689 | hypothetical protein NBO_27g0020 | 2.28 | 1.78E-04 | 20.10 | 5.58 | -0.65 |
| gi\|326565308 | 40S ribosomal protein S9 | 1.14 | 2.09E-04 | 21.56 | 9.99 | -0.73 |
| gi\|326578181 | 60S ribosomal protein L36e | 1.54 | 2.36E-04 | 11.12 | 10.72 | -0.81 |
| gi\|326559144 | 40S ribosomal protein S8-B | 8.51 | 3.63E-04 | 19.03 | 10.27 | -0.83 |
| gi\|484856077 | transcriptional activator | 0.23 | 4.96E-04 | 32.82 | 9.87 | -1.28 |
| gi\|326578117 | 60S ribosomal protein L24 | 1.59 | 5.52E-04 | 10.28 | 11.09 | -0.61 |
| gi\|326578097 | 60S ribosomal protein L4 | 1.36 | 5.67E-04 | 37.84 | 9.74 | -0.40 |
| gi\|484856380 | Glyceraldehyde-3-phosphate dehydrogenase 2, partial | 0.83 | 6.21E-04 | 33.38 | 6.26 | -0.29 |
| gi\|484854054 | hypothetical protein NBO_462g0009 | 2.95 | 9.17E-04 | 18.37 | 7.84 | -0.85 |
| gi\|259511816 | spore wall protein 30 | 1.71 | 9.24E-04 | 32.09 | 8.11 | -0.10 |
| gi\|484856153 | hypothetical protein NBO_48g0007 | 0.42 | 9.40E-04 | 34.81 | 5.42 | -0.73 |
| gi\|484853834 | RING finger protein 121 | 0.48 | 9.42E-04 | 39.97 | 8.35 | 0.25 |
| gi\|484854163 | hypothetical protein NBO_444g0006 | 0.25 | 1.05E-03 | 40.66 | 4.15 | -1.25 |
| gi\|484852887 | Nuclear pore complex protein Nup98-Nup96 | 1.36 | 1.17E-03 | 71.62 | 9.27 | -0.48 |
| gi\|326578137 | 60S ribosomal protein L15 | 1.87 | 1.32E-03 | 24.07 | 10.77 | -0.84 |
| gi\|484852311 | hypothetical protein NBO_1190g0001 | 1.99 | 1.45E-03 | 24.87 | 5.42 | -1.65 |
| gi\|326574320 | 60S ribosomal protein L3, partial | 1.25 | 1.49E-03 | 76.43 | 9.04 | -0.43 |
| gi\|484856628 | Actin | 1.27 | 1.57E-03 | 42.19 | 5.83 | -0.19 |
| gi\|484852777 | hypothetical protein NBO_979g0001 | 1.83 | 1.99E-03 | 21.02 | 9.03 | -0.43 |
| gi\|484856559 | hypothetical protein NBO_29g0019 | 1.55 | 2.02E-03 | 24.38 | 4.82 | -0.77 |
| gi\|326578131 | 60S ribosomal protein L8 | 1.29 | 2.19E-03 | 25.84 | 9.91 | -0.42 |
| gi\|212292371 | histone H3_2 | 1.35 | 2.25E-03 | 16.96 | 10.50 | -0.53 |
| gi\|484854980 | Proteasome subunit alpha type7-like protein | 0.79 | 2.94E-03 | 26.07 | 4.98 | -0.16 |
| gi\|484855499 | 26S protease regulatory subunit 6A | 0.69 | 2.99E-03 | 8.30 | 7.79 | -0.23 |
| gi\|484856688 | hypothetical protein NBO_27g0019, partial | 1.57 | 3.34E-03 | 30.97 | 6.25 | -0.71 |
| gi\|484857497 | hypothetical protein NBO_7g0059 | 0.23 | 3.57E-03 | 30.62 | 4.82 | -0.45 |
| gi\|326566477 | 40S ribosomal protein S24-A | 1.30 | 3.57E-03 | 15.13 | 10.16 | -1.04 |
| gi\|484857234 | Flap endonuclease 1-A | 0.18 | 3.71E-03 | 14.78 | 8.71 | -0.41 |
| gi\|484852349 | Muscle M-line assembly protein unc-89 | 1.89 | 4.03E-03 | 36.80 | 9.45 | -1.60 |
| gi\|484857303 | NUDIX hydrolase | 1.36 | 4.27E-03 | 17.28 | 7.79 | -0.01 |
| gi\|484856166 | Tubulin alpha chain | 1.29 | 4.41E-03 | 49.21 | 5.48 | -0.31 |
| gi\|484856721 | Glutamate NMDA receptor-associated protein 1 | 2.81 | 4.71E-03 | 38.67 | 7.06 | -0.93 |
| gi\|484855746 | T-complex protein 1 subunit epsilon | 0.90 | 4.72E-03 | 58.39 | 5.12 | -0.16 |
| gi\|484854187 | U6 snRNA-associated Sm-like protein LSm1 | 0.50 | 5.11E-03 | 11.73 | 6.59 | -0.22 |
| gi\|484854236 | hypothetical protein NBO_428g0001 | 0.72 | 5.16E-03 | 11.96 | 8.97 | -0.54 |
| gi\|484852837 | Polar tube protein 1 | 2.77 | 5.47E-03 | 40.47 | 5.82 | -0.05 |
| gi\|484857462 | hypothetical protein NBO_7g0021 | 0.77 | 5.69E-03 | 29.31 | 9.14 | 0.66 |
| gi\|484855755 | hypothetical protein NBO_66g0042 | 0.69 | 5.97E-03 | 53.43 | 4.73 | -0.90 |
| gi\|484852219 | hypothetical protein NBO_1230g0002 | 0.38 | 6.25E-03 | 26.55 | 8.90 | -0.77 |
| gi\|484857383 | Enolase | 0.90 | 6.76E-03 | 46.32 | 6.13 | -0.24 |
| gi\|484853316 | hypothetical protein NBO_704g0001 | 0.64 | 6.90E-03 | 9.09 | 5.34 | 0.10 |
| gi\|484854492 | cytosol aminopeptidase, partial | 0.82 | 7.12E-03 | 27.42 | 5.88 | -0.55 |
| gi\|255098781 | 60S ribosomal protein L10 | 1.57 | 7.17E-03 | 24.89 | 10.22 | -0.51 |
| gi\|484855718 | PRE-mRNA splicing helicase | 2.22 | 7.20E-03 | 72.19 | 5.99 | -0.23 |
| gi\|484857001 | hypothetical protein NBO_16g0043 | 0.53 | 7.69E-03 | 10.98 | 9.64 | -1.85 |
| gi\|484855581 | actin-like 53kDa protein | 0.57 | 7.69E-03 | 29.02 | 5.80 | -0.19 |
| gi\|484856491 | Elongation factor 1-alpha | 1.25 | 7.71E-03 | 34.17 | 8.61 | -0.30 |
| gi\|484854534 | hypothetical protein NBO_376g0009 | 2.28 | 8.57E-03 | 10.05 | 9.82 | -0.60 |
| gi\|597956417 | septin 3, partial | 1.15 | 8.81E-03 | 28.72 | 4.97 | -0.71 |
| gi\|484854317 | GPN-loop GTPase 2 | 1.30 | 9.73E-03 | 30.66 | 4.60 | -0.23 |
| gi\|484857275 | hypothetical protein NBO_10g0012 | 0.90 | 9.92E-03 | 44.76 | 8.98 | -0.83 |
| gi\|484852727 | SEC18-like vesicular fusion protein | 0.94 | 1.06E-02 | 77.41 | 5.92 | -0.24 |
| gi\|484851912 | hypothetical protein NBO_1373g0002 | 0.80 | 1.06E-02 | 24.05 | 8.36 | -0.58 |
| gi\|326566073 | 40S ribosomal protein S30 | 1.55 | 1.06E-02 | 7.10 | 11.31 | -1.28 |
| gi\|326570713 | 60S ribosomal protein L32 | 1.56 | 1.09E-02 | 16.02 | 10.32 | -0.58 |
| gi\|484857316 | Polar tube protein 3 | 1.21 | 1.14E-02 | 150.32 | 6.29 | -0.71 |
| gi\|484853415 | DNA-directed RNA polymerases I and III subunit RPAC1 | 0.44 | 1.18E-02 | 35.83 | 5.96 | -0.25 |
| gi\|484857043 | VIP36-like vesicular integral membrane protein, partial | 1.21 | 1.23E-02 | 43.10 | 5.76 | -0.34 |
| gi\|484856052 | Ribose-5-phosphate isomerase A | 1.38 | 1.35E-02 | 13.15 | 7.72 | -0.08 |
| gi\|484855243 | hypothetical protein NBO_149g0001 | 0.77 | 1.37E-02 | 27.67 | 9.07 | -1.12 |
| gi\|484853857 | T-complex protein 1 subunit gamma | 0.61 | 1.56E-02 | 58.41 | 8.28 | -0.22 |
| gi\|484852862 | hypothetical protein NBO_937g0001, partial | 0.67 | 1.56E-02 | 33.10 | 9.38 | -0.38 |
| gi\|484855809 | Serine/threonine-protein-phosphatase PP2A-2 catalytic subunit | 1.34 | 1.56E-02 | 34.40 | 5.14 | -0.19 |
| gi\|134285548 | unknown | 0.45 | 1.60E-02 | 31.33 | 9.03 | -0.84 |
| gi\|484854029 | DNA-directed RNA polymerase III subunit RPC2 | 0.22 | 1.81E-02 | 126.27 | 7.97 | -0.23 |
| gi\|484857797 | Muskelin | 1.24 | 1.85E-02 | 50.98 | 6.25 | -0.67 |
| gi\|212292373 | histone H3_3 | 1.33 | 1.93E-02 | 16.13 | 10.62 | -0.40 |
| gi\|484857000 | hypothetical protein NBO_16g0042 | 0.21 | 1.98E-02 | 12.20 | 5.88 | -1.89 |
| gi\|326578091 | 60S ribosomal protein L23 | 1.21 | 2.01E-02 | 16.09 | 10.06 | -0.53 |
| gi\|484852292 | aminopeptidase p-like protein | 1.12 | 2.07E-02 | 9.04 | 6.08 | -0.83 |
| gi\|484856047 | Heterogeneous nuclear ribonucleoprotein D-like protein, partial | 1.32 | 2.11E-02 | 50.22 | 8.28 | -1.26 |
| gi\|134285544 | unknown | 1.27 | 2.11E-02 | 8.98 | 9.51 | 0.85 |
| gi\|484855144 | proteasome subunit alpha type-7 | 0.82 | 2.21E-02 | 25.05 | 5.40 | -0.22 |
| gi\|255098773 | 40S ribosomal protein S26 | 1.35 | 2.41E-02 | 11.90 | 10.17 | -0.70 |
| gi\|484855476 | BOS1-like vesicular transport protein | 0.42 | 2.47E-02 | 23.71 | 8.63 | -0.46 |
| gi\|484857384 | hypothetical protein NBO_9g0004 | 2.07 | 2.51E-02 | 17.57 | 8.71 | 1.22 |
| gi\|484856492 | Glycolipid 2-alpha-mannosyltransferase | 1.35 | 2.52E-02 | 38.49 | 7.54 | -0.28 |
| gi\|484852603 | hypothetical protein NBO_1056g0001 | 1.27 | 2.57E-02 | 32.54 | 5.20 | -1.14 |
| gi\|484855909 | hypothetical protein NBO_61g0002 | 2.56 | 2.61E-02 | 13.42 | 8.32 | -0.66 |
| gi\|484856109 | Proteasome component Y13 | 0.86 | 2.67E-02 | 25.71 | 5.12 | -0.24 |
| gi\|326578153 | 60S ribosomal protein L37a | 1.68 | 2.88E-02 | 10.16 | 10.53 | -0.57 |
| gi\|326578125 | 60S ribosomal protein L18a | 1.10 | 2.93E-02 | 21.08 | 9.78 | -0.59 |
| gi\|484855633 | phosphatidylinositol transfer protein | 0.58 | 2.93E-02 | 24.92 | 7.82 | -0.68 |
| gi\|326559176 | 40S ribosomal protein S18 | 0.88 | 2.94E-02 | 13.16 | 10.72 | -0.57 |
| gi\|484852458 | hypothetical protein NBO_1120g0001 | 0.34 | 2.95E-02 | 12.32 | 5.91 | -1.87 |
| gi\|484856072 | Ubiquitin thioesterase otubain-like protein | 0.37 | 3.01E-02 | 25.45 | 5.01 | -0.47 |
| gi\|484854858 | Cleavage and polyadenylation specificity factor subunit 3, partial | 0.51 | 3.02E-02 | 60.95 | 5.76 | -0.21 |
| gi\|484852144 | Exosome complex exonuclease RRP40 | 1.23 | 3.21E-02 | 28.52 | 9.43 | 0.08 |
| gi\|484852962 | 1-acyl-sn-glycerol-3-phosphate acyltransferase 4, partial | 0.55 | 3.31E-02 | 32.40 | 9.32 | 0.07 |
| gi\|484852834 | leucyl tRNA synthetase | 0.46 | 3.34E-02 | 8.40 | 4.89 | -0.41 |
| gi\|484851771 | hypothetical protein NBO_1468g0001 | 0.82 | 3.47E-02 | 30.14 | 4.86 | -0.62 |
| gi\|484856070 | Pre-mRNA cleavage complex II protein Clp1 | 0.75 | 3.52E-02 | 43.15 | 6.47 | -0.10 |
| gi\|484852631 | hypothetical protein NBO_1043g0001 | 0.81 | 3.71E-02 | 23.93 | 8.22 | -0.30 |
| gi\|484855859 | Exportin-1 | 0.71 | 3.74E-02 | 10.17 | 4.31 | -0.39 |
| gi\|484854706 | Bifunctional polynucleotide phosphatase /kinase | 0.80 | 3.75E-02 | 39.34 | 9.13 | -0.44 |
| gi\|484855557 | hypothetical protein NBO_76g0001 | 0.71 | 3.78E-02 | 67.34 | 6.28 | -0.59 |
| gi\|484853402 | Transcription-associated recombination protein | 1.76 | 3.87E-02 | 6.99 | 4.53 | 0.41 |
| gi\|484852432 | hypothetical protein NBO_1135g0001 | 1.33 | 3.89E-02 | 28.32 | 6.74 | -0.71 |
| gi\|326567624 | 40S ribosomal protein S3aE | 0.89 | 3.89E-02 | 26.41 | 9.96 | -0.54 |
| gi\|484856832 | hypothetical protein NBO_23g0005 | 1.16 | 3.96E-02 | 22.15 | 7.56 | -0.75 |
| gi\|484856037 | hypothetical protein NBO_55g0015, partial | 1.30 | 3.98E-02 | 66.73 | 5.25 | -0.06 |
| gi\|484855041 | hypothetical protein NBO_224g0001 | 1.30 | 4.19E-02 | 89.54 | 7.41 | -1.21 |
| gi\|484855975 | putative spore wall protein 9 | 0.87 | 4.22E-02 | 42.83 | 8.39 | -0.41 |
| gi\|484856756 | putative spore wall protein 4 | 1.33 | 4.25E-02 | 50.02 | 4.94 | 0.00 |
| gi\|326573099 | 60S ribosomal protein L22 | 0.73 | 4.27E-02 | 13.11 | 9.46 | -0.49 |
| gi\|484857273 | Putative deoxyribonuclease TATDN1 | 0.56 | 4.28E-02 | 31.15 | 5.45 | -0.38 |
| gi\|484857433 | hypothetical protein NBO_8g0039 | 0.47 | 4.33E-02 | 38.01 | 5.10 | -0.80 |
| gi\|484854273 | hypothetical protein NBO_420g0001 | 1.62 | 4.38E-02 | 25.65 | 10.11 | -1.13 |
| gi\|484856456 | Glycylpeptide N-tetradecanoyltransferase 2 | 0.84 | 4.48E-02 | 41.92 | 5.85 | -0.45 |
| gi\|484854047 | hypothetical protein NBO_462g0002 | 1.47 | 4.57E-02 | 17.91 | 5.23 | -0.24 |
| gi\|484855228 | Ubiquitin | 1.22 | 4.58E-02 | 17.72 | 8.48 | -0.80 |
| gi\|326578177 | 60S ribosomal protein L5, partial | 0.53 | 4.62E-02 | 34.65 | 5.10 | -0.50 |
| gi\|484855153 | hypothetical protein NBO_175g0001 | 1.27 | 4.65E-02 | 22.05 | 4.62 | -0.21 |
| gi\|484854698 | hypothetical protein NBO_354g0001 | 0.48 | 4.66E-02 | 50.68 | 5.51 | -0.55 |
| gi\|326578107 | 60S ribosomal protein L34 | 2.70 | 4.74E-02 | 22.59 | 10.49 | -0.29 |
| gi\|484857458 | Splicing factor, arginine/serine-rich 10 | 0.71 | 4.84E-02 | 54.58 | 4.94 | -1.66 |
| gi\|484857318 | Pre-mRNA 3'-end-processing factor FIP1, partial | 0.53 | 4.84E-02 | 28.67 | 5.10 | -0.93 |
| gi\|484854334 | hypothetical protein NBO_413g0001 | 1.29 | 4.92E-02 | 64.53 | 8.72 | -1.27 |
| gi\|484857593 | hypothetical protein NBO_6g0083 | 0.56 | 4.92E-02 | 11.54 | 6.58 | -0.43 |
