# Supplementary figures and images for: Quantitative Proteomic Analysis of Germination of Nosema bombycis Spores under Extremely Alkaline Conditions
Source: Front Microbiol. 2016 Sep 21;7:1459. doi: 10.3389/fmicb.2016.01459 (PMC5030232; doi:10.3389/fmicb.2016.01459)

**S6 Dataset. KEGG pathway diagrams for the 127 significantly changed proteins**


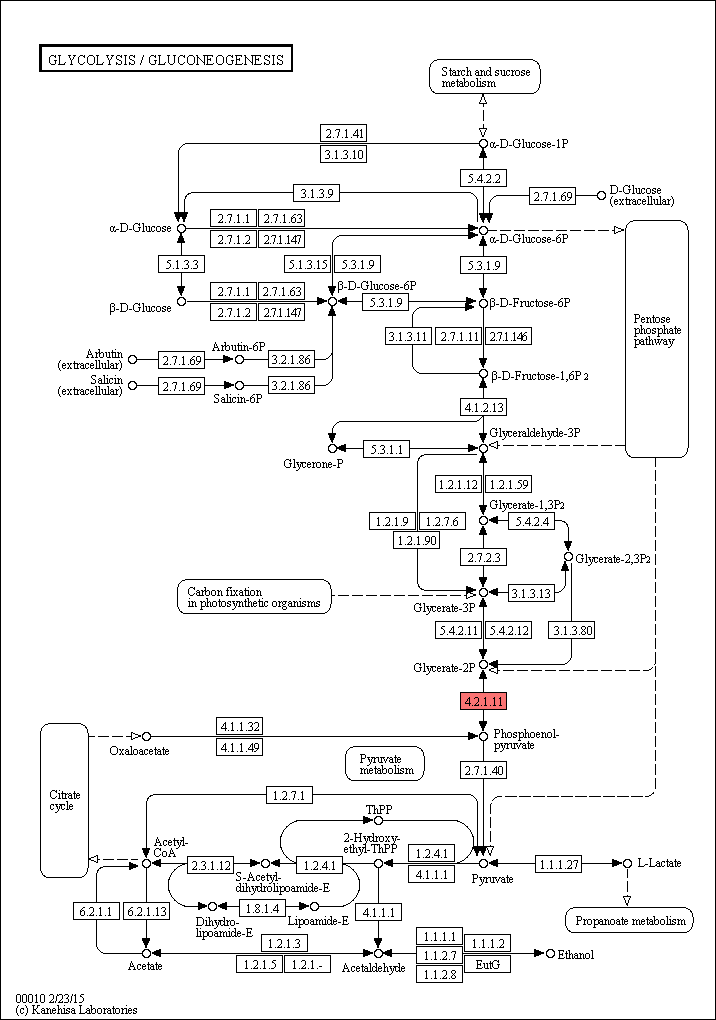

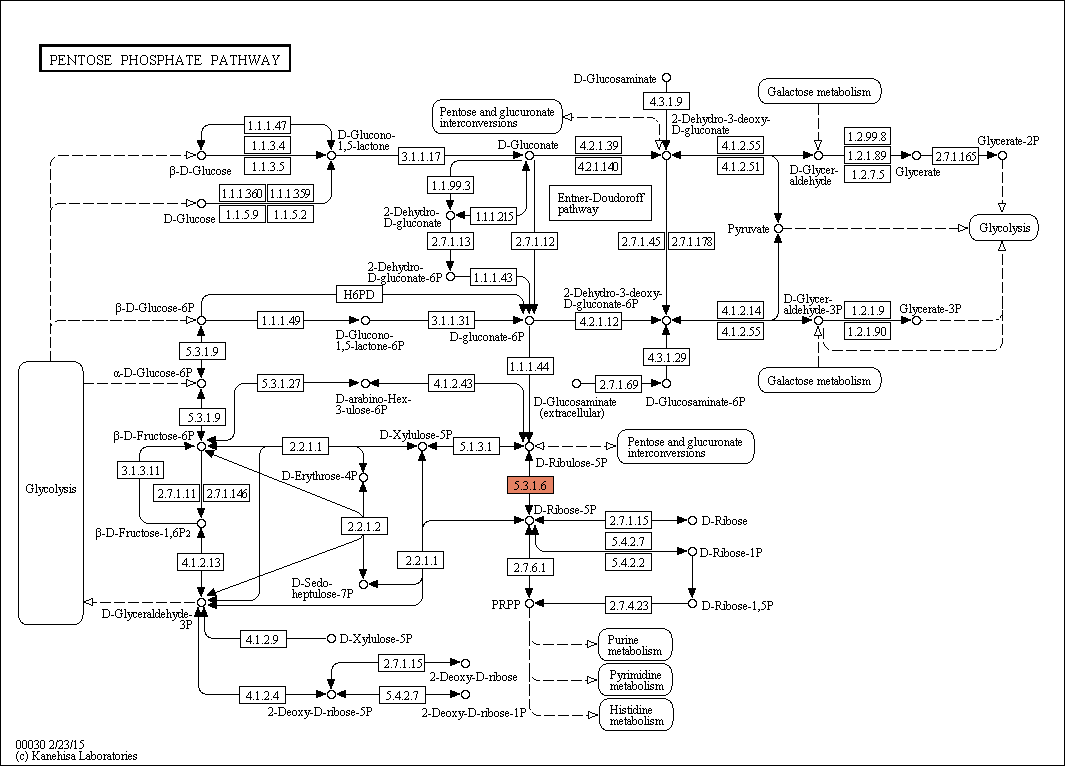

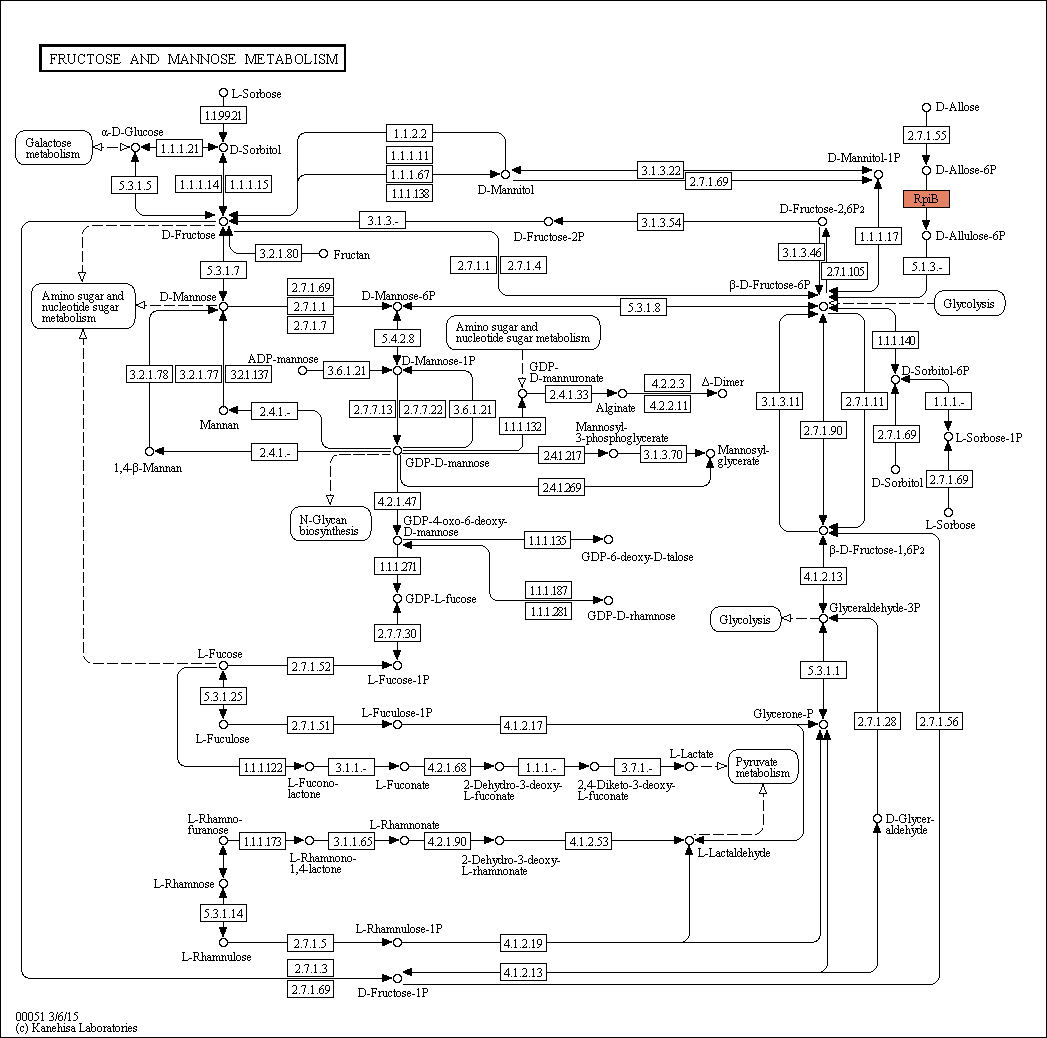


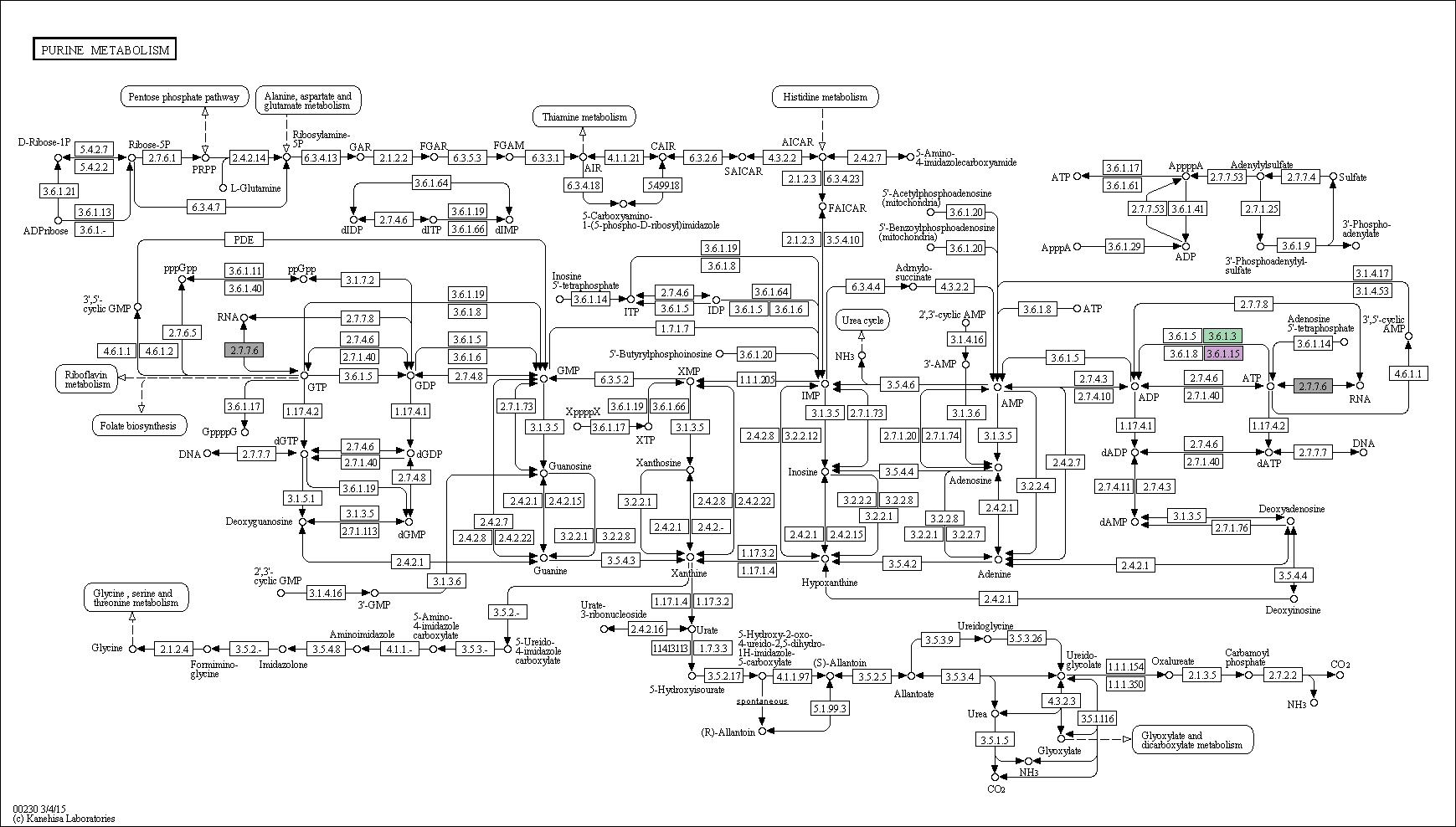


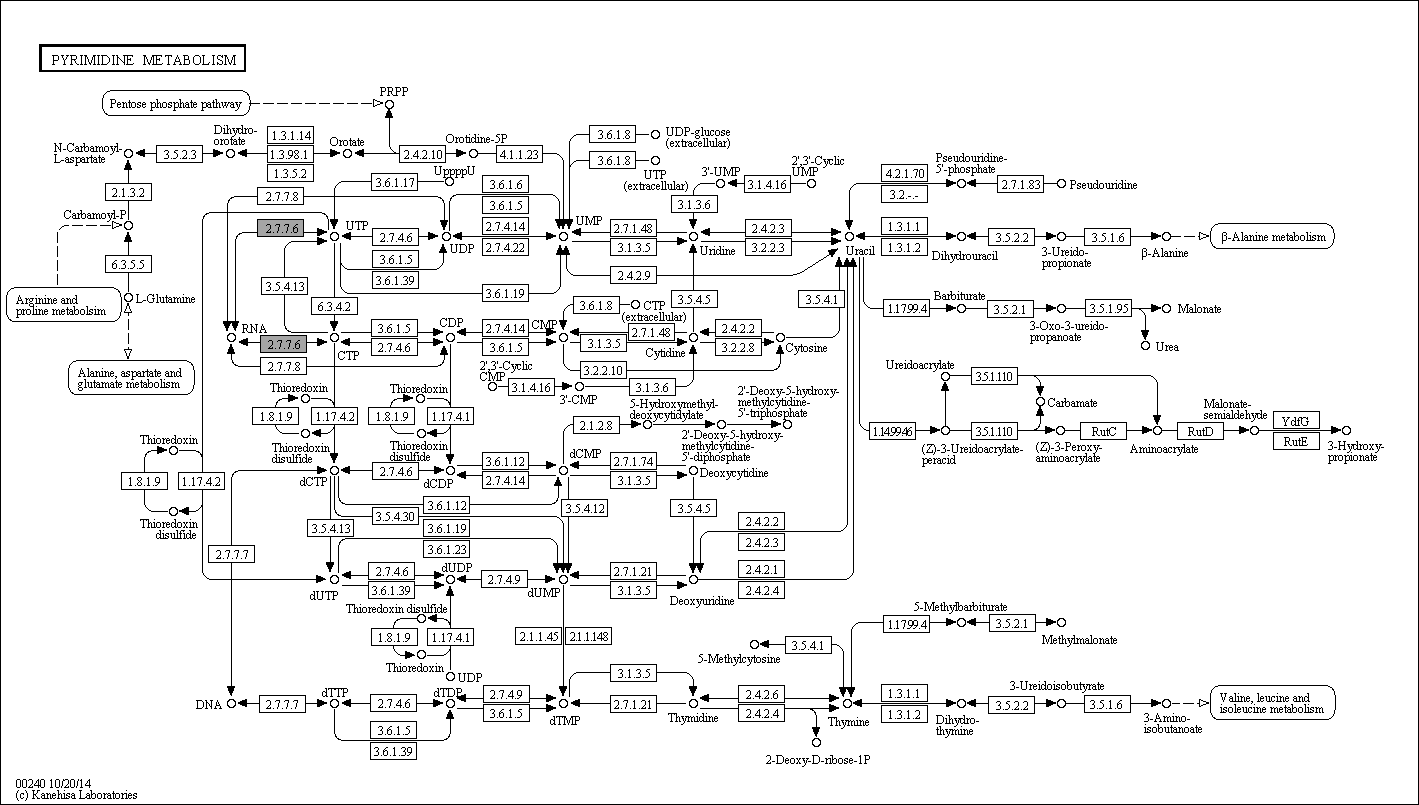


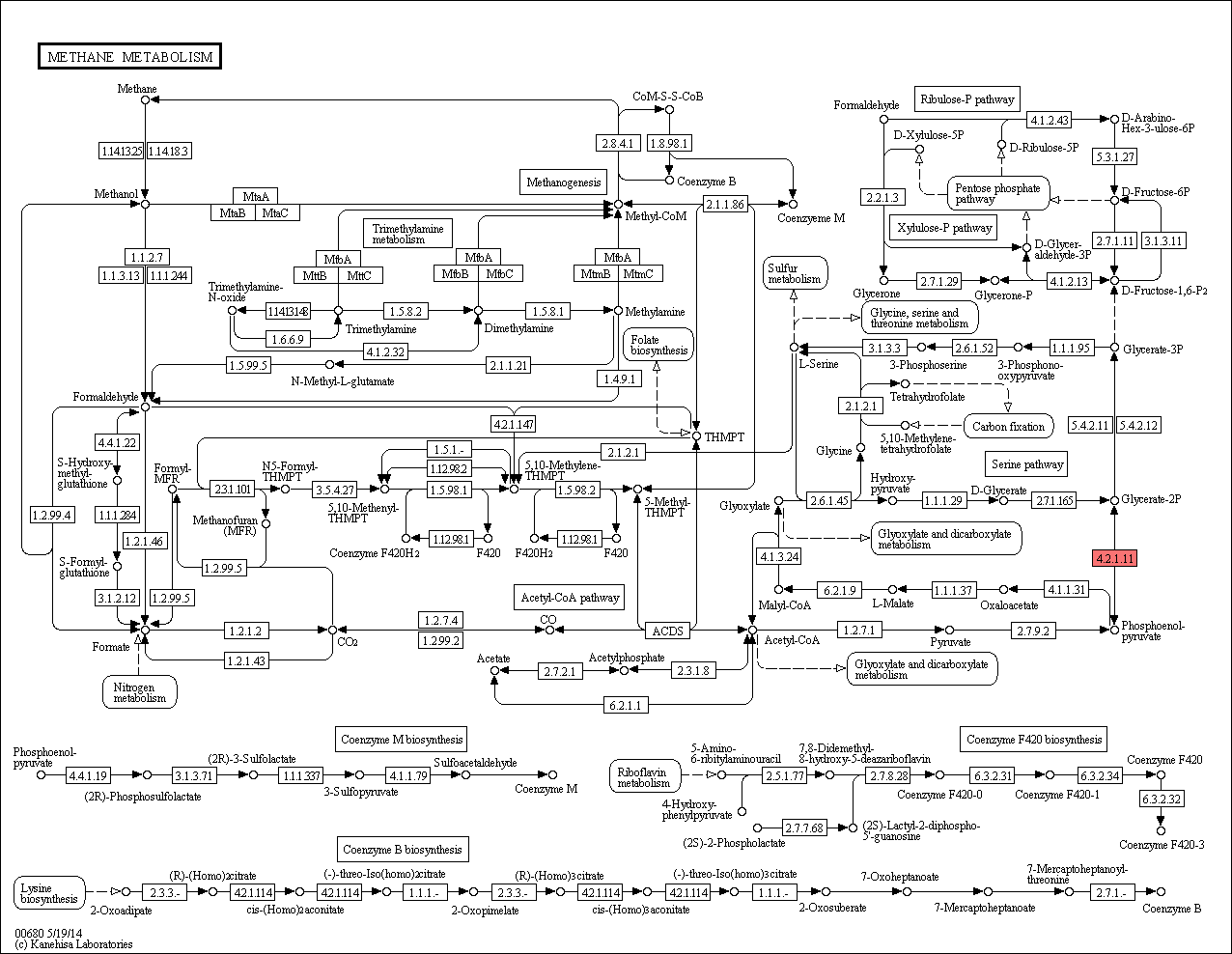


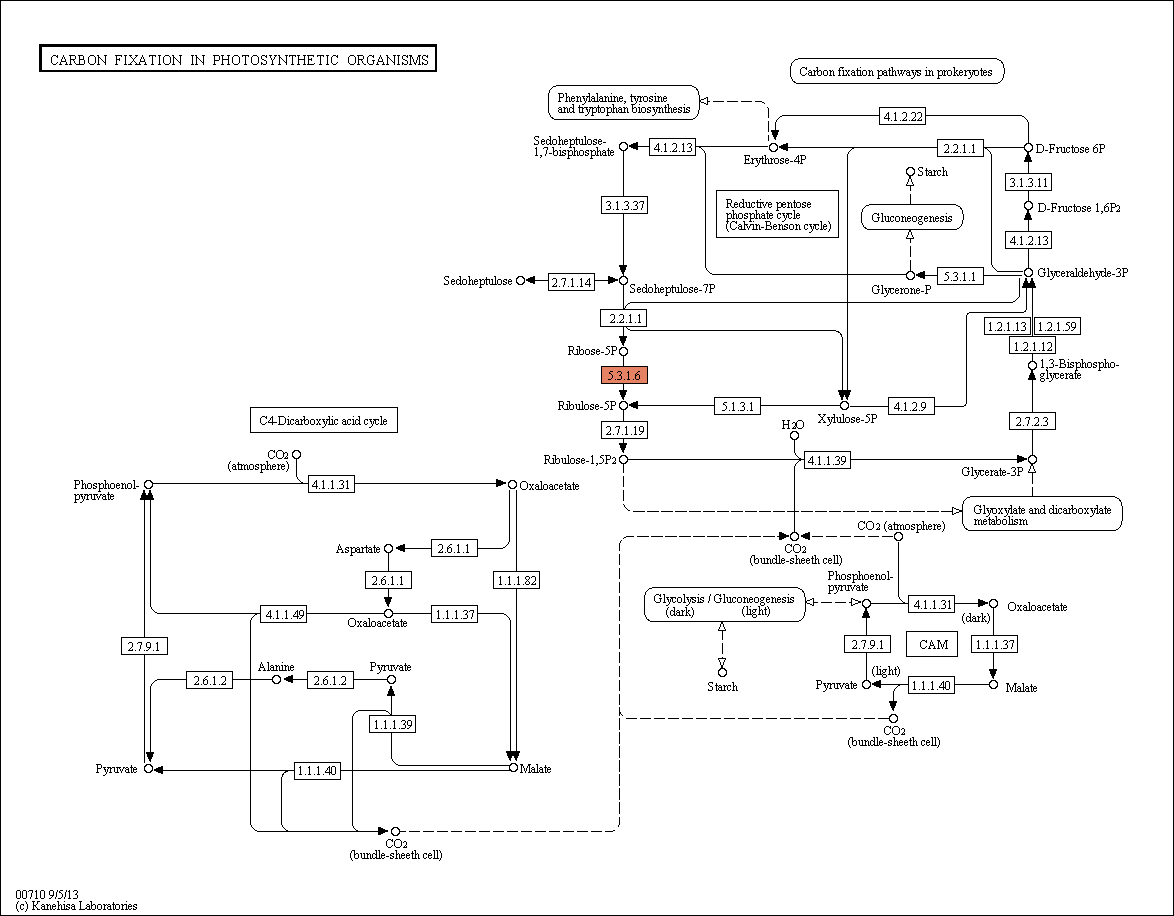


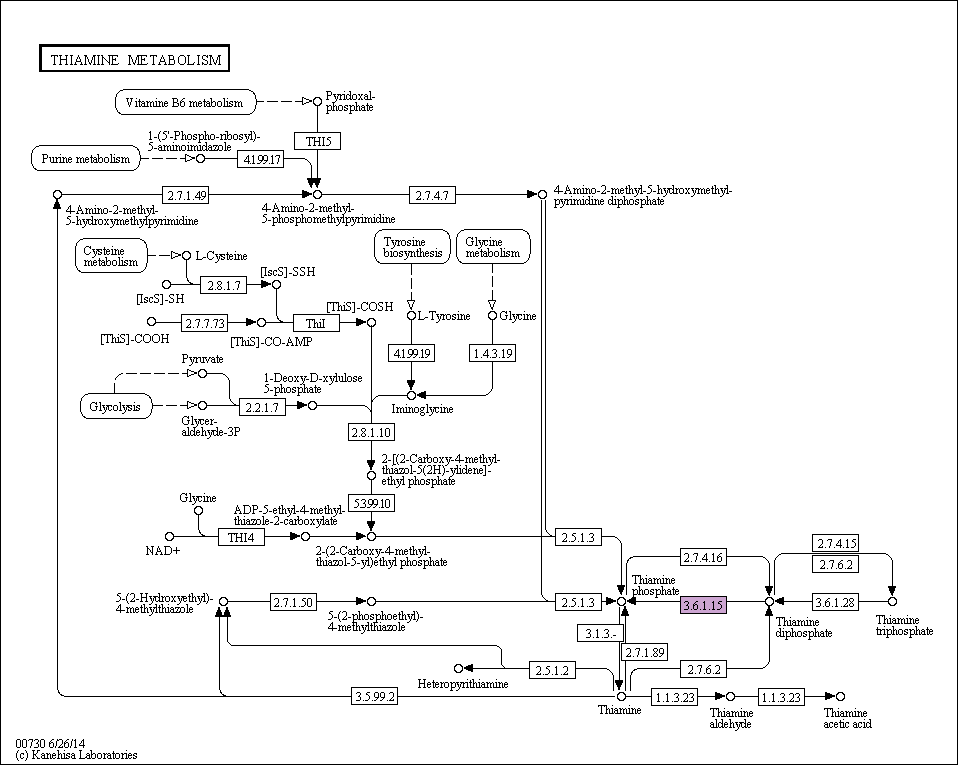


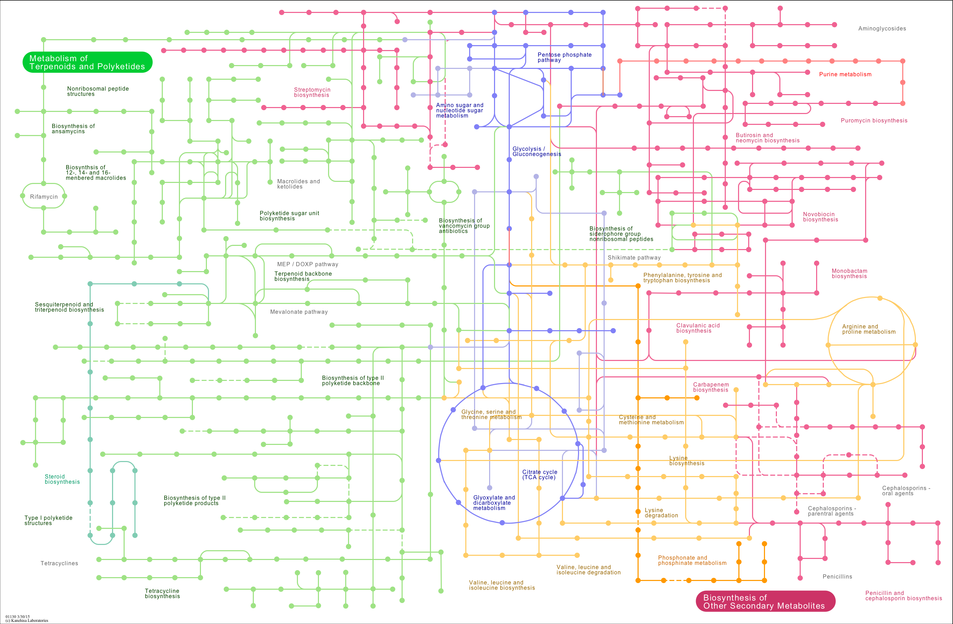

Supplement: Dataset S6 — KEGG pathway diagrams for the 127 significantly changed proteins. [file DataSheet1.docx]
